# Engineered poly(A)-surrogates for translational regulation and therapeutic biocomputation in mammalian cells

**DOI:** 10.1038/s41422-023-00896-y

**Published:** 2024-01-04

**Authors:** Jiawei Shao, Shichao Li, Xinyuan Qiu, Jian Jiang, Lihang Zhang, Pengli Wang, Yaqing Si, Yuhang Wu, Minghui He, Qiqi Xiong, Liuqi Zhao, Yilin Li, Yuxuan Fan, Mirta Viviani, Yu Fu, Chaohua Wu, Ting Gao, Lingyun Zhu, Martin Fussenegger, Hui Wang, Mingqi Xie

**Affiliations:** 1 International Institutes of Medicine, The Fourth Affiliated Hospital, Zhejiang University School of Medicine, Yiwu, Zhejiang China; 2 Key Laboratory of Growth Regulation and Translational Research of Zhejiang Province, School of Life Sciences, Westlake University, Hangzhou, Zhejiang China; 3 Westlake Laboratory of Life Sciences and Biomedicine, Hangzhou, Zhejiang China; 4 Institute of Basic Medical Sciences, Westlake Institute for Advanced Study, Hangzhou, Zhejiang China; 5 College of Life Sciences, Zhejiang University, Hangzhou, Zhejiang China; 6 Department of Biology and Chemistry, College of Science, National University of Defense Technology, Changsha, Hunan China; 7 School of Life Sciences, Fudan University, Shanghai, China; 8 Research Center of Biological Computation, Zhejiang Laboratory, Hangzhou, Zhejiang China; 9 Department of Biosystems Science and Engineering, ETH Zurich, Mattenstrasse 26, CH-4058 Basel, Switzerland; 10 Faculty of Science, University of Basel, Mattenstrasse 26, CH-4058 Basel, Switzerland; 11 School of Engineering, Westlake University, Hangzhou, Zhejiang China

**Keywords:** Riboswitches, Targeted therapies

## Abstract

Here, we present a gene regulation strategy enabling programmable control over eukaryotic translational initiation. By excising the natural poly-adenylation (poly-A) signal of target genes and replacing it with a synthetic control region harboring RNA-binding protein (RBP)-specific aptamers, cap-dependent translation is rendered exclusively dependent on synthetic translation initiation factors (STIFs) containing different RBPs engineered to conditionally associate with different eIF4F-binding proteins (eIFBPs). This modular design framework facilitates the engineering of various gene switches and intracellular sensors responding to many user-defined trigger signals of interest, demonstrating tightly controlled, rapid and reversible regulation of transgene expression in mammalian cells as well as compatibility with various clinically applicable delivery routes of in vivo gene therapy. Therapeutic efficacy was demonstrated in two animal models. To exemplify disease treatments that require on-demand drug secretion, we show that a custom-designed gene switch triggered by the FDA-approved drug grazoprevir can effectively control insulin expression and restore glucose homeostasis in diabetic mice. For diseases that require instantaneous sense-and-response treatment programs, we create highly specific sensors for various subcellularly (mis)localized protein markers (such as cancer-related fusion proteins) and show that translation-based protein sensors can be used either alone or in combination with other cell-state classification strategies to create therapeutic biocomputers driving self-sufficient elimination of tumor cells in mice. This design strategy demonstrates unprecedented flexibility for translational regulation and could form the basis for a novel class of programmable gene therapies in vivo.

## Introduction

Precise regulation of (trans)gene activities is critical for achieving optimal efficacy and safety of gene- and cell-based therapies. In recent years, many trigger-inducible gene regulation systems have been developed for use in mammalian cells, enabling expression of target genes to be controlled by various user-defined exogenous signals and/or intracellular states.^1^ For example, optogenetics can provide traceless, non-invasive, long-distance communication between electronic devices and biological systems, allowing portable electronics — such as smartphones — to regulate cellular activities.^2^ Human cells can also be programmed to sense extracellular disease markers such as blood glucose^3^ or pH^4^ or internal signals such as redox states^5^ or miRNA signatures^6^ and respond by initiating customized therapeutic actions, thus enabling the development of disease-specific control devices that provide automated diagnosis and treatment.

Most gene regulation systems reported so far act at the transcriptional level, and therefore have relatively slow sense-and-response dynamics.^7^ In contrast, regulation systems operating at the level of protein translation are faster-acting and have attracted increasing interest in recent years.^8^ Translation-based systems would also be applicable to sense a wider variety of intracellular signals, since transcription-based sensors are inherently limited to the detection of signals transduced into the nucleus.^9^ However, the engineering of inducible translational regulation in mammalian cells has been hampered by the limited information on “convergent” molecular mechanisms of translation initiation.^10^ As a result, many translational regulation devices developed to date are inhibitory rather than inducible in nature,^11,12^ where ligand responsiveness is primarily achieved through prevention of the interruption and/or termination of mRNA activity.^13–16^ In contrast, engineering of inducible protein translation, which would allow a trigger signal to directly activate translation of target mRNA, has remained a challenging task.

In eukaryotic cells, protein translation is initiated when a preinitiation complex consisting of a 40S ribosome and initiation factors (eIFs) is recruited to the untranslated region (UTR) at the guanine-rich 5′-cap of mature mRNA molecules that have been exported to the cytoplasm.^10,17^ Cooperative activity by cap-binding protein eIF4E, RNA helicase eIF4A, central scaffolding protein eIF4G and helicase enhancers eIF4B and eIF4H then subsequently triggers RNA unwinding, ribosome attachment and codon scanning.^10^ It is well known that the 3′-poly-adenine (poly(A)) tail enhances mRNA stability in living cells, but the underlying mechanism is still a subject of debate.^10,18^ Nevertheless, numerous studies support a “closed-loop” model in which poly(A)-binding protein (PABP) functions as another canonical eIF capable of simultaneously binding both poly(A) and eIF4G to induce a circularized mRNA configuration that is favorable for mRNA scanning, ribosome recycling and protein translation.^10,19^ To engineer trigger-inducible translational devices, incorporation of RNA-binding protein (RBP)-specific aptamers into the UTRs of target gene mRNA is a popular starting point, as it enables site-specific recruitment of RBP-containing regulatory proteins designed to control mRNA stability or eIF4E recruitment.^9,20,21^ However, current approaches have not particularly considered the closed-loop model in the control of translation, and perhaps for this reason, many systems show only limited efficacy in vivo,^9,13,15,16,20–26^ which would limit their clinical relevance.

In this work, we show that genetically encoded replacement of the natural poly(A) signal with synthetic RBP-specific aptamers plays a decisive role in overcoming the poor regulatory performance that hampered earlier attempts to achieve efficient translational control in mammalian cells and in vivo, allowing initiation of translation to depend strictly on the presence of synthetic translational initiation factors (STIFs) engineered to bind both the 3′-aptamer region and the eIF4F-complex at the 5′-cap. This in turn sets the stage for a new regulation strategy that enables systematic engineering of gene switches and intracellular sensors that respond to a multitude of biomedically relevant control signals of interest. For example, we show that a custom-designed gene switch triggered by the FDA-approved drug grazoprevir could effectively control insulin expression and restore glucose homeostasis in diabetic mice, while remaining compatible with various DNA- and RNA-centered gene therapy delivery strategies currently used in the clinic. In addition, we show that a unique advantage of the STIF architecture is the ability to custom-develop genetically encoded sensors to detect various subcellularly (mis)localized proteins in a quantitative manner, such as the BCR-ABL fusion protein of chronic myelogenous leukemia (CML). In line with this, we describe various designs of intracellular protein sensors with the unique potential to either displace or substantially enhance state-of-the-art cell-state classifier circuits to create next-generation “therapeutic biocomputers” for future precision therapies. By illustrating complexity and specificity issues that could become relevant in a clinical context, we eventually demonstrate self-sufficient elimination of cancer cells in mice mediated by such protein-responsive gene therapies. This work describes an undiscovered strategy for translational regulation, adds intracellular protein sensing to the portfolio of cell-state classification strategies and could accelerate the clinical translation of various programmable gene therapies.

## Results

### Design and construction of functional poly(A)-surrogates for translational regulation in mammalian cells

**Fig. 1:**
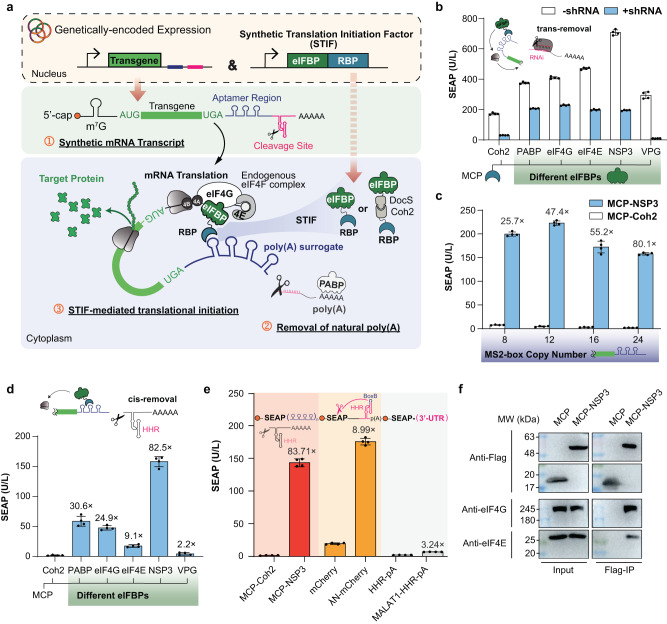
Translational regulation mediated by engineered poly(A)-surrogates. **a** Working scheme of STIF-dependent gene expression control. Translation of reporter mRNA with genetically modified 3′-UTR containing an RBP-specific aptamer region and an shRNA- or HHR-mediated cleavage site for preprogrammed poly(A) removal depends on the presence of STIFs consisting of different RBPs fused to different eIFBPs. Because STIFs are designed to mimic the role of natural PABP in simultaneously binding target mRNA and one member of the eIF4F complex (i.e., eIF4G, eIF4E, eIF4A, eIF4B, etc.) to form a “closed-loop” mRNA configuration and activate translation, poly(A) removal is essential to achieve effective and autonomous (trans)gene control and to avoid putative crosstalk with endogenous PABP-driven processes. **b** STIF-mediated translation of SEAP mRNA containing shRNA-216-cleavable poly(A). HEK-293 cells were co-transfected with a SEAP expression vector containing 8 tandem MCP-specific MS2-box repeats (P_hCMV_-SEAP-(MS2)_8_-BS_shRNA-216_-pA; pSL1331), an shRNA-216 expression vector (P_hU6_-shRNA-216; pSL4, 100 ng) and expression vectors for different STIF variants (PABP-MCP, pSL1315; eIF4G-MCP, pSL154; MCP-eIF4E, pSL1316; MCP-NSP3, pSL95; MCP-VPg, pLYL47) or an MCP-Coh2 protein incapable of initiating translation (pSL674, negative control). SEAP levels in culture supernatants were quantified at 48 h post transfection. Data are means ± SD of *n* = 4 independent experiments. **c** Correlation between aptamer region size and STIF-mediated SEAP expression. HEK-293 cells were transfected with SEAP expression vectors containing different tandem repeats of the MCP-specific MS2-box aptamer ((MS2-box)_8_, pSL515; (MS2-box)_12_, pSL1284; (MS2-box)_16_, pSL516; (MS2-box)_24_, pSL468) and constitutive expression vectors for either MCP-NSP3 (ON; pSL95) or an MCP-Coh2 protein incapable of binding eIF4F (OFF; pSL674). SEAP expression in culture supernatants was scored at 48 h post transfection. Data are presented as means ± SD; *n* = 4 individual experiments. **d** STIF-mediated translation of SEAP mRNA containing HHR-cleavable poly(A). HEK-293 cells were co-transfected with a SEAP expression vector containing 24 tandem MCP-specific MS2-box repeats (P_hCMV_-SEAP-(MS2 box)_24_-HHR-pA; pSL468) and expression vectors for different STIF variants (PABP-MCP, pSL1315; eIF4G-MCP, pSL154; MCP-eIF4E, pSL1316; MCP-NSP3, pSL95; MCP-VPg, pLYL47) or an MCP-Coh2 protein incapable of initiating translation (pSL674, negative control). SEAP levels in culture supernatants were quantified at 48 h post transfection. Data are shown as means ± SD of *n* = 4 independent experiments. **e** Translational regulation by MCP-based STIF and other state-of-the-art regulation strategies. For STIF-mediated translation (red), HEK-293 cells were co-transfected with a SEAP expression vector containing 24 tandem MCP-specific MS2-box repeats (reporter: P_hCMV_-SEAP-(MS2-box)_24_-HHR-pA, pSL468) and constitutive expression vectors for MCP-Coh2 (OFF; pSL674) or MCP-NSP3 (ON; pSL95). For translational regulation by ligand-inhibited ribozyme activity (orange; inspired by ref. ^20^), HEK-293 cells were co-transfected with a SEAP expression vector containing bacteriophage λ N-Peptide (λN)-repressible HHR placed upstream of poly(A) (reporter: P_SV40_-SEAP-HHR-pA, pMX116) and constitutive expression vectors for λN-mCherry (ON; pSA776) or mCherry (OFF; pQX183). For translational regulation by modulation of 3′-UTR stability (gray; inspired by ref. ^22^), HEK-293 cells were transfected with expression vectors for SEAP mRNA containing no poly(A) (OFF; pMX116, SEAP-HHR-pA) or the lncRNA MALAT1 in the 3′-UTR (ON; pLZ323, SEAP-MALAT1-HHR-pA). SEAP levels in culture supernatants were scored at 48 h post transfection. Data are shown as means ± SD; *n* = 4 independent experiments. **f** STIFs activate target gene translation by associating with the endogenous eIF4F complex. HEK-293 cells were transfected with a SEAP expression vector containing 24 tandem MCP-specific MS2-box repeats (pSL468) and expression vectors for either 3×FLAG-tagged MCP (pSL1084) or MCP-NSP3 (pSL1083) before one lysate fraction was immunoprecipitated at 48 h post transfection. Target proteins in lysate fractions before (input) and after immunoprecipitation (Flag-IP) were detected with anti-FLAG, anti-eIF4G and anti-eIF4E antibodies. SEAP levels in culture supernatants were measured at 48 h post transfection (Supplementary information, Fig. S3e). Bars represent means ± SD, and filled circles show individual results; numbers above bars are fold changes (**b**–**e**).

Following the “closed-loop” model, we considered that manipulating the mRNA circularization process would be an attractive accession point to achieve effective regulation over translational initiation.^10,18^ Thus, we first created expression vectors for a synthetic mRNA transcript that harbors a control region consisting of RBP-specific aptamers in the 3′-UTR (Fig. 1a). Circularization and translation of this mRNA transcript would then depend on the presence of a STIF that mimics natural PABP function by simultaneously binding the aptamer-based control region and any one member of the preinitiation complex. Specifically, we engineered various STIF constructs by fusing the archaeal ribosomal protein L7Ae^11^ to different eIF4F-binding proteins (eIFBPs), such as human PABP, eIF4G and eIF4E, as well as rotaviral non-structural protein 3 (NSP3)^27^ and caliciviral VPg.^25^ Although most eIFBPs significantly upregulated reporter gene expression from human placental secreted alkaline phosphatase (SEAP) mRNA harboring L7Ae-specific C/D-box aptamers, basal expression of the reporter gene was too high to meet biomedical requirements (Supplementary information, Fig. S1a, white columns). We hypothesized that this high background activity could be attributed to the presence of the natural poly(A) tail located downstream of the synthetic aptamer region, which may attract endogenous factors (such as native PABP) to form a closed-loop configuration and activate translation even in the absence of STIFs (Fig. 1a). Therefore, to enhance STIF-dependent regulation, we placed shRNA-binding sites (Supplementary information, Fig. S1b) between the aptamer region and the poly(A) region to remove the poly(A) tail through ectopic co-expression of a specific shRNA (Fig. 1a). As expected, SEAP expression from poly(A)-deficient mRNA showed markedly reduced background levels and increased STIF-dependency (Supplementary information, Fig. S1a, blue columns), and the relative fold changes between basal STIF-independent expression and STIF-mediated upregulation could be further fine-tuned by increasing the number of aptamer repeats placed downstream of the protein-coding region (Supplementary information, Fig. S1c). Thus, this aptamer region is reminiscent of a poly(A)-surrogate mediating strict STIF-dependent mRNA translation, with genetically encoded poly(A)-excision emerging as seminal for effective (trans)gene control by enabling “escape” from endogenous PABP-mediated processes (Supplementary information, Fig. S1d). Notably, the size of this engineered poly(A)-surrogate, reflecting the number of tandem aptamer repeats placed in the 3′-UTR, appears to have an impact on delaying mRNA decay in mammalian cells (Supplementary information, Fig. S1e), and any aptamer-specific protein binding to such region may confer increased stability on the target mRNA (Supplementary information, Fig. S1e,f).

Next, we engineered one-component expression vectors for poly(A)-deficient mRNA by replacing *trans*-acting shRNA-binding sites with *cis*-acting hammerhead ribozyme (HHR) motifs^20^ (Supplementary information, Fig. S1g), which should trigger spontaneous self-excision of the natural poly(A) signal either before or immediately after nuclear mRNA export. In this configuration, L7Ae-NSP3 was the STIF candidate showing the highest fold-induction in the regulation of L7Ae-specific SEAP mRNA translation (Supplementary information, Fig. S1h). Importantly, control experiments showing that non-specific eIFBP overexpression cannot activate SEAP expression further confirmed STIF-specificity of translational activation (Supplementary information, Fig. S2a). The STIF-dependent regulation scheme is also modular in terms of compatibility with different RBP:aptamer systems, as was demonstrated by engineering other STIF variants in which L7Ae was replaced by bacteriophage-derived MS2 coat protein (MCP).^28^ For example, fusion of MCP to the same eIFBP candidates (Fig. 1b; Supplementary information, Fig. S1a) can also activate gene expression from poly(A)-deficient mRNA with different tandem repeats of cognate MS2-box motifs (Fig. 1c), with MCP-NSP3 showing the highest induction folds between basal and activated states following HHR-mediated poly(A)-removal (Fig. 1d). Again, binding of MCP-containing proteins to cognate poly(A)-surrogates might increase the stability of corresponding MS2-box-containing mRNA (Supplementary information, Fig. S2b) without significantly affecting endogenous gene expression capacities of the mammalian host cell (Supplementary information, Fig. S2c). Interestingly, larger sizes of MS2-box-based poly(A)-surrogates appear to have a lesser impact on constitutive translation efficiency when directly compared to C/D-box-based counterparts (Supplementary information, Fig. S2d, e). Thus, the combination of MCP-NSP3 and SEAP mRNA harboring 24 MCP-specific MS2-box repeats (P_hCMV_-SEAP-(MS2-box)_24_-HHR-pA) was established as the preferred configuration for translational regulation (Fig. 1e, red), which displayed highly dynamic ranges of fold change favorable over our experimental implementations of state-of-the-art approaches that capitalize on inhibition of ribozyme activity (Fig. 1e, orange; inspired by ref. ^20^), modulation of 3′-UTR stability (Fig. 1e, gray; inspired by ref. ^22^) or natural 5′-cap-independent translation (Supplementary information, Fig. S2f, g; inspired by refs. ^21,23–25^). MCP-NSP3 could also efficiently regulate a variety of other reporter genes, such as those encoding firefly luciferase (Supplementary information, Fig. S3a, b) or enhanced green fluorescence protein (EGFP) (Supplementary information, Fig. S3c, d). In addition, we experimentally confirmed that NSP3-containing STIFs did associate with the eIF4F complex underlying the closed-loop model for activation of STIF-specific translation (Fig. 1f; Supplementary information, Fig. S3e), that NSP3 showed only marginal non-specific binding to endogenous RNA (Supplementary information, Fig. S3f), that STIF-specific translation was indeed dependent on endogenous eIF4G and eIF4E proteins (Supplementary information, Fig. S3g) and that endogenous eIF4F expression levels were principally sufficient to drive near-maximal STIF-specific translation (Supplementary information, Fig. S3h, i). Thus, for the purpose of autonomous transgene control in human cells involving minimal interference with endogenous cellular processes, we chose NSP3 as the eIFBP moiety for all further engineering studies.

### A framework for designing trigger-inducible STIFs

**Fig. 2:**
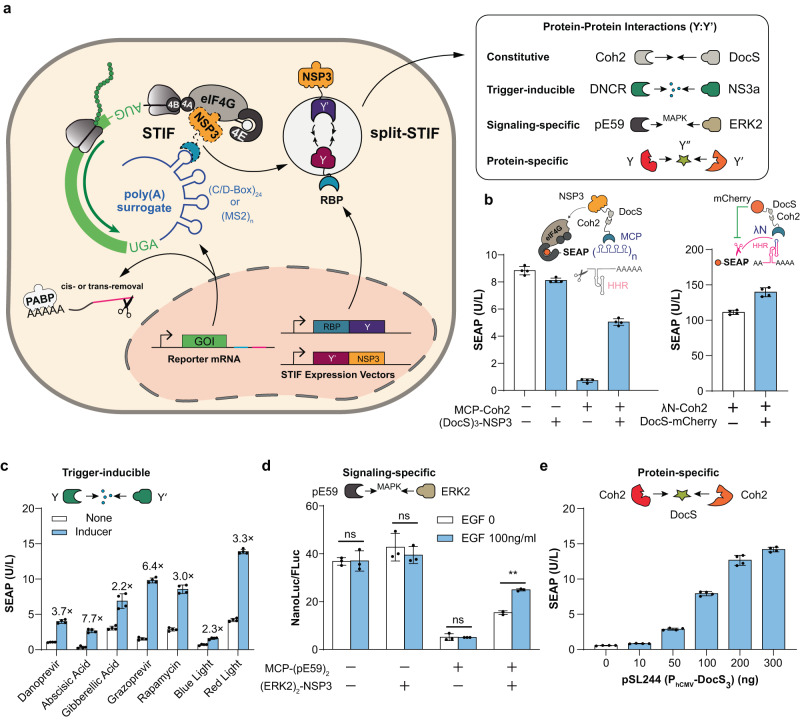
Tailoring stimulus-responsive gene expression using a multipartite STIF design framework. **a** Translational regulation by protein–protein interaction (PPI)-mediated STIF reconstitution. Bipartite STIF systems with NSP3 and RBP domains split into two independent proteins allow flexible incorporation of different PPI systems to regulate conditional STIF assembly and translation of RBP-specific mRNA. **b** Compatibility of STIF-mediated translational regulation with split-complementation approaches. Left, HEK-293 cells were co-transfected with a SEAP expression vector containing 24 tandem MCP-specific aptamer repeats (pSL468) and different combinations of constitutive expression vectors for MCP-NSP3 (pSL674) and (DocS)_3_-NSP3 (pSL86). Right, HEK-293 cells were co-transfected with a SEAP expression vector containing bacteriophage λ N-Peptide (λN)-repressible HHR placed upstream of poly(A) (pMX116) and constitutive expression vectors for λN-Coh2 (pSL334) and DocS-mCherry (pSL1099). For (–) conditions, pcDNA3.1(+) was transfected instead of expression vectors. SEAP levels in culture supernatants were scored at 48 h post transfection. Data are shown as means ± SD; *n* = 4 independent experiments. **c** Regulation through trigger-inducible STIF reconstitution. For danoprevir-inducible SEAP translation, HEK-293 cells were co-transfected with pSL468 and constitutive expression vectors for MCP-NS3a (pSL497) and DNCR-NSP3 (pLZ72). For abscisic acid-inducible SEAP translation, HEK-293 cells were co-transfected with pSL468 and constitutive expression vectors for ABI-MCP (pPW22) and (PYL)_3_-NSP3 (pPW4). For gibberellic acid-inducible SEAP translation, HEK-293 cells were co-transfected with pSL468 and constitutive expression vectors for MCP-GID (pPW23) and GAI-NSP3 (pPW2). For grazoprevir-inducible SEAP translation, HEK-293 cells were co-transfected with pSL468 and constitutive expression vectors for MCP-NS3a (pSL497) and GNCR-NSP3 (pYF3). For rapamycin-inducible SEAP translation, HEK-293 cells were co-transfected with a SEAP expression vector containing poly(A)-surrogates with 16 tandem MCP-specific MS2-box repeats (pSL516) and constitutive expression vectors for MCP-FRB (pSL1097) and FKBP-NSP3 (pSL1098). For blue light-inducible SEAP translation, HEK-293 cells were co-transfected with pSL516 and constitutive expression vectors for MCP-CIB1 (pSL1096) and Cry2-NSP3 (pSL71). For red light-inducible SEAP translation, HEK-293 cells were co-transfected with pSL516 and constitutive expression vectors for MCP-(Aff6_V18FΔN_)_4_ (pSL917) and DrBPhP-NSP3 (pSL901). SEAP levels in culture supernatants were scored at 48 h after addition of the corresponding inducers (danoprevir, 0.5 µM; abscisic acid, 100 µM; gibberellic acid, 100 µM; grazoprevir, 0.5 µM; rapalog, 0.1 µM) or at 48 h after exposure to blue light (450 nm; ON, 30 s at 5 mW/cm^2^; OFF, 30 s) or at 24 h after exposure to red light (660 nm, constantly 1 W/m^2^). Data are shown as means ± SD; *n* = 4 independent experiments. **d** Genetically encoded signaling-specific sensors employing phosphorylation-dependent STIF reconstitution. HEK-293 cells were co-transfected with a constitutive FLuc expression vector (pYW99), an expression vector for NanoLuc-mRNA containing MCP-specific poly(A)-surrogates (P_hCMV_-NanoLuc-P2A-mCherry-(MS2-box)_24_-HHR-pA, pSL683) and different combinations of constitutive expression vectors for MCP-(pE59)_2_ (pSL637) and (ERK2)_2_-NSP3 (pSL189) before cultivation in cell culture medium containing 2% fetal bovine serum (FBS) (v/v). Luciferase levels in culture supernatants were quantified at 48 h after the addition of 0 or 100 ng/mL recombinant human EGF. For (–) conditions, pcDNA3.1(+) was transfected instead of expression vectors. Data are presented as means ± SD of relative luciferase activity (NanoLuc/FLuc); *n* = 3 individual experiments. **e** Genetically encoded protein sensors employing protein association-dependent STIF reconstitution. HEK-293 cells were co-transfected with pSL468 and constitutive expression vectors for MCP-(Coh2)_3_ (pSL1080), (Coh2)_3_-NSP3 (pSL243) and (DocS)_3_ (different amounts of pSL244). SEAP levels in culture supernatants were scored at 48 h post transfection. Data are shown as means ± SD of *n* = 4 independent experiments. Bars represent means ± SD, and filled circles show individual results; numbers above bars are fold changes (**b**–**e**). ns, not significant; **P < 0.01.

To exploit the modularity of STIF-dependent translational regulation for a flexible design of gene expression systems responding to various trigger signals of interest, we created bipartite STIF systems in which the NSP3 and RBP domains are split into two independent proteins that must be conditionally assembled via different combinations of protein–protein interactions (Fig. 2a). Thus, we first confirmed that single STIF can indeed be separated into a split architecture and that constitutive protein association pairs (such as Coh2:DocS,^29^ Bcl-XL:LD3^30^ and NS3a(H1):ANR^31^) can mediate STIF reconstitution and activate translation of RBP-specific mRNA (Fig. 2b; Supplementary information, Figs. S2a, S3j). Next, we incorporated various trigger-inducible dimerization systems into the split STIF framework, including rapamycin-inducible FKBP:FRB,^32^ danoprevir-inducible DNCR2:NS3a,^33^ grazoprevir-inducible GNCR1:NS3a,^33^ gibberellic acid-inducible GAI:GID,^34^ abscisic acid-inducible ABI:PYL,^35^ blue light-inducible Cry2:CIB1^36^ and red light-inducible DrBphP:Aff6_V18FΔN_ protein–protein interactions.^37^ Although rapamycin and blue light failed to trigger SEAP expression in experimental setups that involve L7Ae-specific STIF systems (Supplementary information, Fig. S4a), the other systems all yielded functional gene switches (Fig. 2c). Notably, while poly(A)-surrogates comprising 24 tandem aptamer repeats usually account for the highest fold-induction (Fig. 1c; Supplementary information, Figs. S1c, S4a), red light- and rapamycin-inducible gene switches based on MCP showed the best performance when regulating translation of target gene mRNA that contains 16 MS2-box motifs (Fig. 2c). Light-inducible gene switches could also be engineered by incorporating the blue light-dependent LaM8-AK47 nanobody^38^ into the STIF framework, thus demonstrating versatility and design flexibility for translational regulation (Supplementary information, Fig. S4b). Protein–protein interactions are also governed by various signal transduction pathways that phosphorylate specific target proteins upon activation.^39^ Thus, to demonstrate feasibility to also engineer genetically encoded signaling-specific sensors, we integrated the ERK2-specific pE59 DARPin system^40^ into the bipartite STIF framework. As pE59 was developed to specifically bind phosphorylated ERK2 during MAPK signaling, individual STIF components would only assemble and activate STIF-specific reporter gene expression in cells showing high MAPK activity (Fig. 2d; Supplementary information, Fig. S4c). Lastly, we showed that STIFs could also be rendered dependent on multipartite protein association events. For example, when each split-STIF component is fused to a different member of a protein heterotrimer system, STIF-dependent translation of poly(A)-deficient mRNA would strictly depend on the presence of the remaining member(s) of the full protein complex. To confirm the applicability of this strategy to engineer intracellular protein sensors, we first showed that bipartite STIF constructs could be engineered on the basis of both MCP-Coh2/DocS-NSP3 (Supplementary information, Fig. S5a) and MCP-DocS/Coh2-NSP3 combinations of bipartite STIF constructs (Supplementary information, Fig. S5b). Then, we showed that STIF components containing three tandem repeats of Coh2 (Supplementary information, Fig. S5c, d) and DocS (Supplementary information, Fig. S5e, f) displayed optimal efficiency for dose-dependent translational initiation of poly(A)-deficient SEAP mRNA (Supplementary information, Fig. S5g, h). Hence, we fused three tandem Coh2 repeats to both the RBP domain (L7Ae or MCP) and the NSP3 domain and used both STIF components MCP-(Coh2)_3_ and (Coh2)_3_-NSP3 as a highly specific “molecular pincer” to detect cytosolic (DocS)_3_ in a dose-dependent manner (Fig. 2e).

### Grazoprevir-triggered gene switch for disease treatments requiring on-demand drug activity

**Fig. 3:**
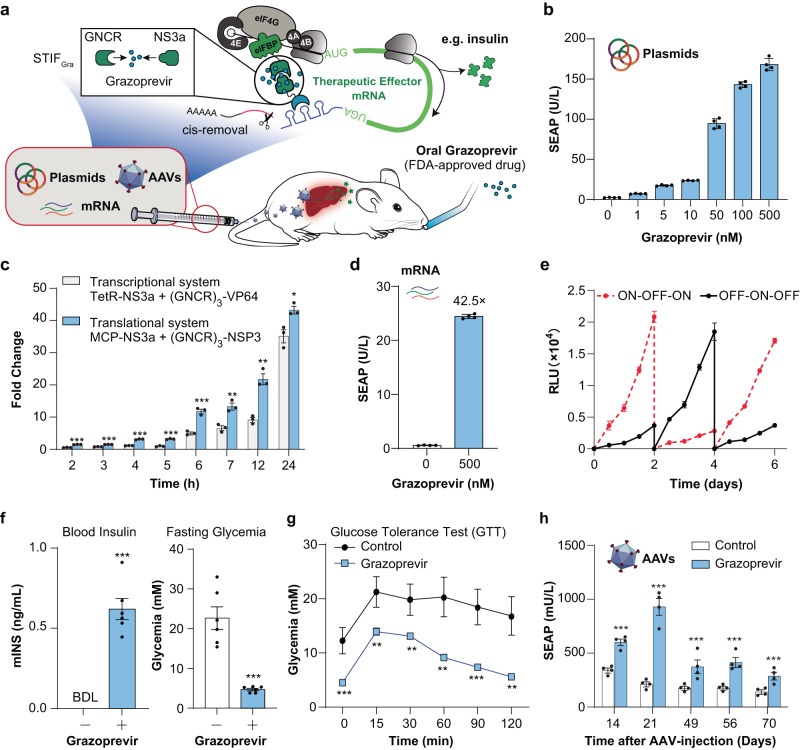
Grazoprevir-inducible gene switch for translational regulation in mammalian cells and gene therapy applications. **a** Regulation of therapeutic transgene expression using the FDA-approved drug grazoprevir. Delivery of the genetic components for GNCR:NS3a-dependent STIF assembly and STIF-specific target mRNA in vivo is compatible with various administration routes of non-integrative gene therapy (e.g., DNA-encoded vectors or formulated mRNA drugs), allowing oral uptake of grazoprevir to trigger in situ production of various therapeutic proteins of interest (e.g., insulin). **b** Dose-dependent grazoprevir-inducible SEAP expression by plasmid DNA-based delivery. HEK-293 cells were co-transfected with plasmids encoding (GNCR)_3_-NSP3 (pSL1032), MCP-(NS3a)_3_ (pSL1042) and SEAP mRNA containing MCP-specific poly(A)-surrogate (pSL468). SEAP levels in culture supernatants were scored at 24 h after addition of different concentrations of grazoprevir. Data are shown as means ± SD of *n* = 4 independent experiments. **c** Kinetics of grazoprevir-inducible SEAP expression. For grazoprevir-inducible translation, HEK-293 cells were co-transfected with plasmids encoding MCP-(NS3a)_3_ (pSL503) and (GNCR)_3_-NSP3 (pLZ74) and pSL468. For grazoprevir-inducible transcription, HEK-293 cells were co-transfected with a TetR-specific SEAP expression vector (tetO_7_-P_hCMVmin_-SEAP-(C/D-box)_24_-(BS_shRNA-216_)_2_-pA, pLZ79) and constitutive expression vectors for TetR-NS3a (pLZ88) and (GNCR)_3_-VP64 (pLZ85). 24 h after transfection, 0 μM or 0.5 μM grazoprevir was added to culture supernatants and SEAP levels were monitored for another 24 h. Data show means ± SD of fold changes calculated by dividing SEAP levels of grazoprevir-treated samples by those of non-grazoprevir-treated samples (*n* = 3 individual experiments). **d** Grazoprevir-inducible SEAP translation by mRNA delivery. HEK-293 cells were (co-)transfected with in vitro-transcribed mRNA encoding for MCP-(NS3a)_3_ (from pSL1085), (GNCR)_3_-NSP3 (from pYW361) and SEAP-(MS2-box)_24_ (from pSL468). SEAP levels in culture supernatants were quantified at 48 h after addition of grazoprevir. Data are shown as means ± SD; *n* = 4 independent experiments. **e** Reversibility of grazoprevir-inducible protein secretion in mammalian cells. HEK-293_LSCCS1_ (stably expressing MCP-(NS3a)_3_, (GNCR)_3_-NSP3 and NanoLuc-mRNA containing 24 tandem MS2-box repeats) were cultivated for 7 days while grazoprevir levels in the culture medium were successively switched between 0 nM and 500 nM. NanoLuc levels were measured every 12 h. The cell density was readjusted to 1 × 10^5^ cells/mL every 2–3 days. **f**, **g** Therapeutic efficacy of oral grazoprevir-inducible insulin expression. Plasmids encoding MCP-(NS3a)_3_, (GNCR)_3_-NSP3 and insulin-mRNA containing MCP-specific poly(A)-surrogate (pSL548/pLZ74/pSL685) were hydrodynamically injected into the tail vein of T1D mice. At 6 h post injection, mice were fed with the first of 3 daily administrations of 3 mg/kg grazoprevir. Fasting glycemia and blood modified insulin (mINS) levels of mice were measured at 20 h after the first grazoprevir administration (**f**). Intraperitoneal glucose tolerance tests (GTTs) were performed at 24 h after the first grazoprevir administration (4 h after quantification of blood insulin shown in **f**) (**g**). Data are presented as means ± SEM; *n* = 6 mice per group. **h** Long-term control of grazoprevir-inducible SEAP production in mice. C57BL/6 mice received intravenous injection of 7 × 10^11^ AAV2/8 particles for constitutive expression of (GNCR)_3_-NSP3, MCP-(NS3a)_3_ and SEAP-mRNA containing MCP-specific poly(A)-surrogate. SEAP production in the bloodstream was monitored over 10 weeks. At 24 h before each measurement, mice were fed with the first of 3 daily administrations of 3 mg/kg grazoprevir. Data are presented as means ± SEM; *n* = 4 mice per group. Bars represent means ± SD or SEM, and filled circles show individual results; numbers above bars are fold changes (**b**–**d**, **f**, **h**). *P < 0.05; **P < 0.01; ***P < 0.001.

After demonstrating the utility of STIF-mediated translational regulation to create various gene switches and sensors, we aimed to build on the grazoprevir-inducible GNCR:NS3a system (Fig. 2c; Supplementary information, Fig. S4a) to engineer a clinically relevant gene switch for long-term therapeutic transgene delivery in vivo (Fig. 3a). Grazoprevir (an FDA-approved drug for treating hepatitis C) is an attractive choice of trigger, because it is bioavailable, non-toxic and metabolically inert, and should be applicable to the regulation of a wide variety of protein therapeutics without interfering with their efficacy in vivo. Systematic optimization studies of a grazoprevir-inducible gene switch based on the GNCR:NS3a system indicated that increasing the number of tandem GNCR (Supplementary information, Fig. S6a) and NS3a repeats (Supplementary information, Fig. S6b) was critical for increasing grazoprevir-dependent gene expression. In accordance with these results, split-STIF constructs each containing three tandem GNCR and NS3a repeats showed the best regulation performance in terms of fold change (Supplementary information, Fig. S6c), dose-dependence (Fig. 3b; Supplementary information, Fig. S6d) and activation kinetics (Fig. 3c). Again, we confirmed that endogenous eIF4G and eIF4E only colocalized with MCP-NS3a or L7Ae-NS3a in the presence of grazoprevir (Supplementary information, Fig. S6e), providing the basis for the closed-loop mRNA configuration model. Notably, the grazoprevir-inducible translational gene switch was significantly faster than a counterpart gene switch operating at the transcriptional level (P_hCMV_-TetR-NS3a-pA, pLZ88; P_hCMV_-(GNCR)_3_-VP64-pA, pLZ85; tetO_7_-P_hCMVmin_-SEAP-(C/D-Box)_24_-pA, pLZ79), while producing comparable absolute expression strengths and induction folds over the entire 48 h experimental timespan (Fig. 3c; Supplementary information, Fig. S6f). Furthermore, we showed that our grazoprevir-inducible translational gene switch is compatible with both DNA- (Fig. 3b, c) and RNA-centered delivery strategies (Fig. 3d). In particular, genetically encoded expression platforms can be advantageous in enabling various durable applications in mammalian cells, such as selection of stable cell lines allowing for reversible sense-and-response dynamics over extended time periods (Fig. 3e) and/or integration of genetic components into AAV-based vectors for various therapeutic purposes in vivo (Supplementary information, Table S2; e.g., pSL446/pSL511/pSL512). In contrast, RNA-only systems have relatively short activity windows upon transfection into mammalian cells (Supplementary information, Fig. S6g).

To examine grazoprevir-regulated treatment efficacy in vivo, we first injected STIF-encoding vectors and STIF-specific SEAP expression vectors into the tail vein of mice. Dose-dependent grazoprevir-inducible transgene expression could be achieved either upon intraperitoneal injection (Supplementary information, Fig. S7a) or oral administration (Supplementary information, Fig. S7b), with an oral dose of 3 mg/kg grazoprevir being sufficient to fully activate the system (Supplementary information, Fig. S7c). Next, using insulin as an exemplary therapeutic output (Supplementary information, Fig. S7d), we tested the treatment potential of the grazoprevir-inducible gene switch in an experimental diabetes mouse model of chemically induced insulin deficiency. Indeed, oral grazoprevir administration effectively restored homeostatic blood insulin (Fig. 3f, left) and glucose (Fig. 3f, right) levels, as well as postprandial glucose metabolism (Fig. 3g) of diabetic mice harboring the genetic components for grazoprevir-inducible insulin translation (therapeutic effector mRNA: P_hCMV_-NanoLuc-P2A-mINS-(MS2-box)_24_-HHR-pA). Furthermore, mice injected with AAV2/8 particles carrying the grazoprevir-inducible translational gene switch maintained the expected profile of regulated protein secretion for at least 10 weeks, indicating the potential for long-term treatment efficacy in vivo (Fig. 3h). Altogether, therapeutic transgene delivery using STIF-mediated translational gene switches is compatible with various clinically approved gene therapy products; it could be administered to patients either using AAV vectors for DNA-encoded treatments, or directly formulated as an in vitro-manufactured mRNA drug (Fig. 3a).

### Genetically encoded protein sensors in mammalian cells

**Fig. 4:**
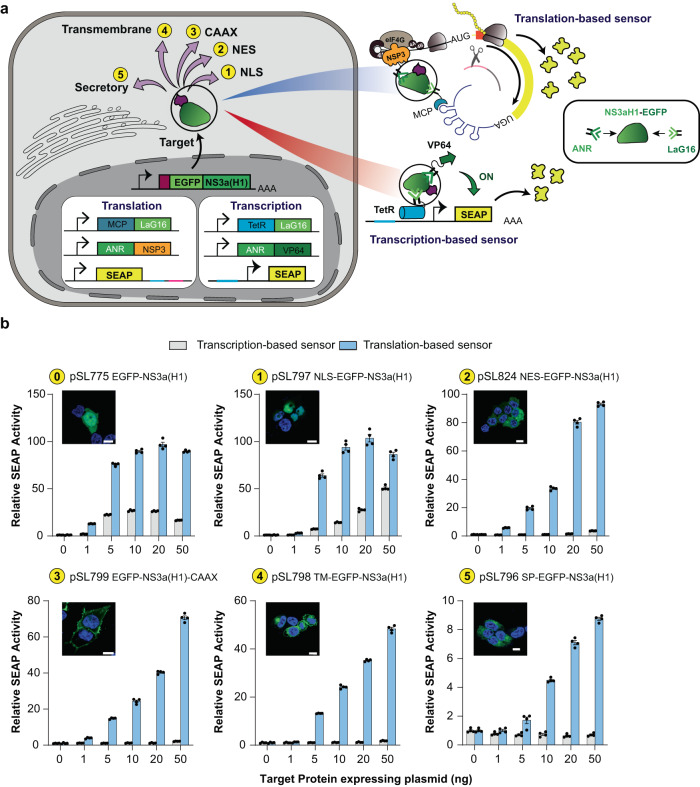
Genetically encoded sensors for intracellular target proteins. **a** Engineering of transcription- and translation-based sensors for detection of differentially localized intracellular proteins. The synthetic target protein EGFP-NS3a(H1) was targeted to different intracellular compartments through fusion with different localization signals (1, NLS (nuclear localization signal); 2, NES (nuclear export signal); 3, CAAX (prenylation motif); 4, transmembrane localization signal; 5, secretory signal peptide) to allow detection with co-expressed genetic sensors engineered on the basis of LaG16 (an EGFP nanobody) and ANR (a peptide motif binding NS3a(H1)). For transcription-based sensing, LaG16 was fused to TetR and ANR was fused to VP64 to allow EGFP-NS3a(H1)-dependent activation of TetR-specific promoters. For translation-based sensing, LaG16 was fused to MCP and ANR was fused to NSP3 to allow EGFP-NS3a(H1)-dependent STIF reconstitution and translation of MCP-specific mRNA. **b** Dose-dependent detection of differentially translocated EGFP-NS3a(H1). HEK-293 cells were co-transfected with plasmids encoding the translation-based EGFP-NS3a(H1) sensor (pSL776/pSL582/pSL468: for expression of MCP-LaG16, (ANR)_8_-NSP3 and SEAP-mRNA with MCP-specific poly(A)-surrogate) or the transcription-based EGFP-NS3a(H1) sensor (pSL834/pSL836/pMF111: for expression of TetR-LaG16, (ANR)_8_-VP64 and a TetR-specific promoter controlling SEAP transcription) and different amounts of constitutive expression vectors for different target proteins (0, native EGFP-NS3a(H1), pSL775; 1, nuclear NLS-EGFP-NS3a(H1), pSL797; 2, cytosolic NES-EGFP-NS3a(H1), pSL824; 3, prenylated EGFP-NS3a(H1)-CAAX, pSL799; 4, membrane-localized TM-EGFP-NS3a(H1), pSL798; 5, secretory SP-EGFP-NS3a(H1), pSL796). At 48 h after transfection, fluorescence images of cellular EGFP signals were acquired (scale bars, 10 μm) and SEAP levels in the culture supernatant were profiled. Data are shown as means ± SD fold change of SEAP activity relative to basal SEAP levels detected with no EGFP-NS3a(H1) expression (0 ng columns). Filled circles represent individual results (*n* = 4 independent experiments).

Although we showed experimentally that the grazoprevir-inducible translational switch operated faster than a transcriptional counterpart, this gain in response speed might not be sufficient to have a dramatic impact in clinical settings, as it still required at least 2 h to achieve statistically significant protein secretion (Fig. 3c). Nevertheless, probably a more striking advantage of translation-based systems is the capability to engineer intracellular sensors for constant detection of differentially localized intracellular signals. To illustrate such a scenario, we first created a synthetic EGFP-NS3a(H1) protein as a model target signal for detection (Fig. 4a). Next, we used our STIF-enabled design blueprint for protein sensors (Fig. 2e) to create highly specific EGFP-NS3a(H1) “pincers” in which MCP is fused to LaG16 (a high-affinity GFP nanobody^41^) and NSP3 is fused to ANR (a peptide motif constitutively binding to NS3a(H1)^31^) (Fig. 4a). In optimal binding efficiency studies, we found that a single LaG16 repeat was sufficient to associate with EGFP (Supplementary information, Fig. S8a), whereas NS3a(H1) detection required multiple tandem ANR-peptide motifs fused to the N-terminus of NSP3 (Supplementary information, Fig. S8b). Thus, constitutive expression of MCP-LaG16, (ANR)_8_-NSP3 and reporter mRNAs containing MCP-specific poly(A)-surrogate (P_hCMV_-SEAP-(MS2-box)_24_-HHR-pA) was established as a highly accurate sensor for intracellular EGFP-NS3a(H1) (Supplementary information, Fig. S8c). Next, we directed EGFP-NS3a(H1) to different subcellular compartments by introducing different signal peptides, and compared the sensing capacity of the translation-based sensor with that of an analogous transcriptional sensor (based on constitutive expression of TetR-LaG16, (ANR)_8_-VP64 and a TetR-specific promoter tetO_7_-P_hCMVmin_) in terms of spatial resolution and signal-to-noise ratio. Whereas the transcription-based sensor was limited to the detection of untagged and nuclear-localized target proteins (Fig. 4b; conditions 0 and 1), the STIF-inspired translational sensor could quantitatively detect all the differentially localized proteins (Fig. 4b; conditions 0–5). Furthermore, both flow cytometry analysis and fluorescence imaging revealed that the MCP-LaG16/(ANR)_8_-NSP3-based protein sensor was highly target-specific when producing mCherry signals from reporter mRNA, showing a strong correlation with EGFP signals produced by overexpressed EGFP-NS3a(H1) (Supplementary information, Fig. S8d), and there was no significant basal mCherry expression in EGFP-deficient cells (Supplementary information, Fig. S8e).

### Protein-driven gene therapies for self-sufficient killing of cancer cells in mice

**Fig. 5:**
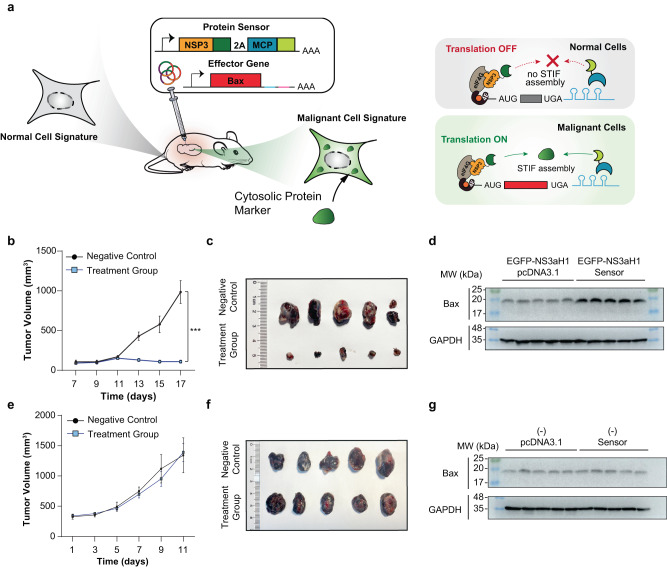
Engineering of target-specific protein sensors driving self-sufficient cancer gene therapies. **a** Validation of therapeutic efficacy using xenograft mouse models. Cell lines with different molecular signatures hypothetically distinguishable by a cytoplasmic target protein were subcutaneously implanted into the lower back of mice and tumors were allowed to grow for 17 days. On day 7, gene circuits engineered for cancer-selective activation of apoptosis were injected into the tumors every 2 days. Tumor size was monitored over the entire experimental timespan. **b**–**d** EGFP-NS3a(H1)-specific activation of apoptosis in mice. Mice harboring subcutaneous B16-F10_EGFP-NS3a(H1)_-derived tumors received local injections of pcDNA3.1(+) (negative control, *n* = 5 mice per group) or plasmid DNA mixture comprising pSL831 (P_hCMV_-mBax-(MS2-box)_24_-HHR-pA), pSL776 (P_hCMV_-MCP-LaG16-pA) and pSL582 (P_hCMV_-(ANR)_8_-NSP3-pA) (treatment group, *n* = 5 mice per group). Daily changes of tumor size were assessed by calculating V = (length × width)^2^/2 (**b**). Tumors were harvested on the final experimental day for weight analysis (**c**) and measurement of Bax protein levels by western blot (**d**). **e**–**g** No activation of apoptosis by a P_hCMV_-driven EGFP-NS3a(H1) sensor in EGFP-NS3a(H1)-deficient tissues. Mice harboring subcutaneous B16-F10-derived tumors received local injections of pcDNA3.1(+) (negative control, *n* = 5 mice per group) or plasmid DNA mixture comprising pSL831/pSL776/pSL582 (treatment group, *n* = 5 mice per group). Daily changes of tumor size were assessed by calculating V = (length × width)^2^/2 (**e**). Tumors were harvested on the final experimental day for weight analysis (**f**) and measurement of Bax protein levels by western blot (**g**).

To showcase treatment potential of STIF-based protein sensors in vivo, we used an experimental model of cancer gene therapy. In brief, we created tumor xenografts by stably expressing EGFP-NS3a(H1) in epithelial B16-F10 cells, exemplifying a malignant cell signature featuring the presence of a specific target protein in the cytosol (Fig. 5a). Seven days after implantation of B16-F10-derived xenografts, mice received daily intratumoral injections of plasmid mixtures encoding for an MCP-LaG16/(ANR)_8_-NSP3-based EGFP-NS3a(H1) sensor driving translation and in situ production of a pro-apoptotic Bax protein. As expected, specific and self-sufficient elimination of EGFP-NS3a(H1)-transgenic tumors was achieved in mice treated with the genetic sensor, whereas mice not receiving the gene therapy (vehicle control) exhibited continuous and rapid tumor growth (Fig. 5b–d). Importantly, no significant activation of apoptosis was observed in mice implanted with native B16-F10 cells not expressing EGFP-NS3a(H1), indicating negligible background Bax expression under a potentially “normal” cell signature (Fig. 5e–g). Importantly, effective protein levels of Bax detected in tumors correlated with the cell lysis profile (Fig. 5d, g), confirming target-specificity of trigger-inducible Bax expression in vivo. For treatment of diseases where highly specific protein markers are available,^42^ such protein sensors would therefore be sufficient to form the basis of an “smartened” gene therapy providing self-sufficient detection and correction of pathologic cell conditions. For example, fusion proteins are hallmarks of many human cancers produced by chromosomal translocations during neoplastic transformation, accounting for a variety of uncontrolled malignant cellular processes.^43^ Indeed, we used our STIF-based design framework and created a sensor for BCR-ABL, a representative cytosolic biomarker of CML^44^ (Supplementary information, Fig. S9a). Strikingly, such STIF-based sensor is highly selective for the fusion gene configuration; while reporter gene expression was dose-dependently activated by the BCR-ABL oncoprotein, the system remained unresponsive to overexpression of native BCR and ABL genes indicative of healthy cell signatures (Supplementary information, Fig. S9b). Furthermore, we showed that overexpression of BCR-ABL indeed triggered assembly of the STIF components MCP-CCmut3 (CCmut3,^45^ a synthetic BCR-specific coiled-coil domain) and ABI(iDab)-NSP3 (ABl(iDab),^46^ an ABL-specific intrabody), providing the basis for activation of STIF-dependent target gene translation (Supplementary information, Fig. S9c). Thus, the STIF-architecture appears to be particularly suited to engineer genetic sensors for various proteins of interest such as cancer-related fusion proteins that include, but evidently not limited to BCR-ABL (Supplementary information, Fig. S9d).

**Fig. 6:**
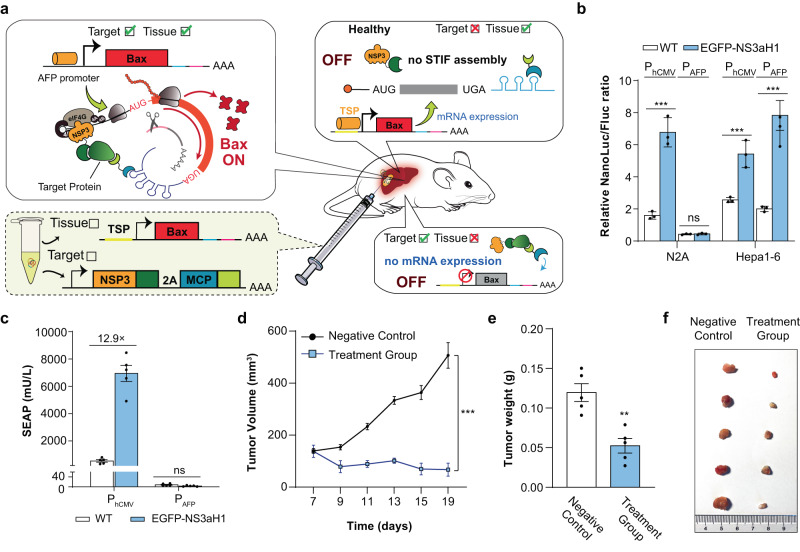
Therapeutic biocomputer for self-sufficient elimination of malignant cells in mice. **a** Design principle of a gene circuit with customized detection algorithms. To precisely distinguish complex cell signatures within heterogenous tissues (e.g., tumors), STIF-based protein sensors with programmable target-specificity could be coupled with TSP driving the production of STIF-specific mRNA for suicide gene expression (e.g., Bax). Upon delivery of such gene circuits into living tissues in vivo, self-sufficient apoptosis is exclusively triggered in cells that meet the criteria of both tissue- (TSP-triggered transcription of poly(A)-deficient STIF-dependent Bax mRNA) AND target-specificity (translation of Bax mRNA only upon STIF-mediated detection of intracellular protein markers). **b** Tissue-specific sensing of EGFP-NS3a(H1) in vitro. Native (WT) or stable EGFP-NS3a(H1)-transgenic N2A and Hepa1-6 cells were co-transfected with P_MusAFP_-driven (pSL813) or constitutive expression vectors for NanoLuc-P2A-mRNA containing an MCP-specific poly(A)-surrogate (pSL683) and constitutive expression vectors for MCP-LaG16 (pSL776), (ANR)_8_-NSP3 (pSL582) and FLuc (pYW99). Luciferase levels were quantified at 48 h post transfection. Data are presented as means ± SD of relative luciferase activity (NanoLuc/FLuc); *n* = 3 individual experiments. **c** Validation of tissue-specific EGFP-NS3a(H1) sensing in mice. Plasmids encoding P_hCMV_-driven (pSL468/pSL582/pSL776) and P_MusAFP_-driven (pSL857/pSL582/pSL776) EGFP-NS3a(H1) sensors and EGFP-NS3a(H1) expression vectors (pSL775) were hydrodynamically injected into the tail vein of C57BL/6 mice. SEAP levels in the bloodstream were measured after 24 h. Mice receiving pcDNA3.1(+) instead of pSL775 were used as negative controls with no hepatic EGFP-NS3a(H1) expression (WT). Data are presented as means ± SEM; *n* = 5 mice per group. **d**–**f** P_MusAFP_- AND EGFP-NS3a(H1)-specific activation of apoptosis in mice. Mice harboring subcutaneous Hepa1-6_EGFP-NS3a(H1)_-derived tumors received local injections of pcDNA3.1(+) (negative control, *n* = 5 mice per group) or plasmid DNA mixture comprising pSL886 (P_MusAFP_-mBax-(MS2-box)_24_-HHR-pA), pSL776 and pSL582 (treatment group, *n* = 5 mice per group). Daily changes of tumor size were assessed by calculating V = (length × width)^2^/2 (**d**). Tumors were harvested and weighed on the final experimental day (**e**, **f**). Bars represent means ± SD, and filled circles show individual results (**b**, **c**, **e**). ns not significant; *P < 0.1; **P < 0.01; ***P < 0.001.

Whereas fusion proteins (e.g., BCR-ABL) are prominent cases where a single intracellular target protein could unambiguously identify pathologic cell states, most diseases typically lack such unique biomarkers. In these cases, a “true” disease-specific cellular signature must be resolved through a combined detection of various subordinate checkpoint signals. To showcase a scenario requiring such multiplexed cell-state detection in a most simplified format, we created a therapeutic biocomputer controlling strict tissue-specific detection of target proteins (Fig. 6a). Although various tissue-specific promoters (TSPs) have been characterized in recent decades with the aim of achieving tissue-specific target gene expression, TSPs alone can hardly meet the requirements of precision targeting in therapeutic settings.^47^ For example, using the alpha-fetoprotein (AFP) promoter P_AFP_ as a model TSP, we could indeed detect non-specific P_AFP_-driven reporter gene expression in AFP-deficient cell lines (Supplementary information, Fig. S10a). Although we experimentally showed that the Bax effector protein could tolerate a relatively high level of basal expression without triggering pre-mature apoptosis at near-homeostatic expression levels (Supplementary information, Fig. S10b, c), more rational cell state identification strategies are urgently required in order to enhance sensing accuracy and increase safety in potential gene therapy settings. Thus, we designed a gene circuit running a two-input sensing algorithm that allows suicide gene expression to only occur in cells that harbor an intracellular target protein (e.g., EGFP-NS3a(H1)) and that reside within specific tissues (e.g., P_AFP_), while tissues not expressing the target protein (i.e., healthy cells) or similar cells from unrelated tissues (e.g., on-target, off-tumor) would remain unaffected (Fig. 6a). To test such a distinction logic in vitro, we first transiently transfected expression vectors for MCP-LaG16, (ANR)_8_-NSP3 and P_AFP_-driven reporter mRNA into neuronal Neuro-2A (N2A) and murine hepatoma-derived Hepa1-6 cells engineered for stable EGFP-NS3a(H1) expression. As expected, only Hepa1-6_EGFP-NS3a(H1)_ cells simultaneously fulfilled both criteria (tissue- AND target-specific gene expression) (Fig. 6b). In mice, while a constitutively expressed EGFP-NS3a(H1) sensor showed significant basal activity in EGFP-NS3a(H1)-deficient tissues, a P_AFP_-driven counterpart remained silent in wild-type (WT) mice expressing hepatic EGFP-NS3a(H1) due to the inactivity of P_AFP_ in healthy livers (Fig. 6c). Finally, using another xenograft model based on Hepa1-6_EGFP-NS3a(H1)_-derived tumors, we demonstrated P_AFP_-driven EGFP-NS3a(H1)-dependent activation of apoptosis in vivo (Fig. 6d–f), with self-sufficient tumor lysis correlating again with effective Bax expression (Supplementary information, Fig. S10d). Thus, these results indicate the potential of how translation-based protein sensors can play a central role in next-generation therapeutic gene circuits providing programmable, broadly adjustable and self-sufficient gene therapies for treatment of many human diseases in the future.

## Discussion

A long-standing goal of synthetic biology-driven cell engineering is the development of sophisticated and programmable therapeutics capable of treating intractable diseases with a precision beyond the scope of current medical technologies. In this context, the aim of our STIF-dependent translational regulation framework is to meet the highest standards of mammalian cell engineering by providing a modular system with the following features: (i) trigger-inducible regulation of transgene expression with high fold changes and fast activation kinetics, (ii) capability of regulating therapeutic protein secretion using clinically licensed small molecules in state-of-the-art gene therapy settings, (iii) robust and seamless integration into complex gene networks consisting of multiple interconnected genetic sensors operating at different stages of gene expression, and most importantly, (iv) capability of logically detecting specific combinations of target compounds of interest independently of their subcellular location. For therapeutic purposes, an ideal translational regulation system should furthermore (v) be compatible with both RNA-only and DNA-based expression to enable flexible adjustment of treatment efficacy, safety and durability according to the medical need, (vi) provide a universal design framework allowing gene expression to be controlled by a broad variety of biomedically relevant input signals and (vii) most evidently, show efficacious performance in vivo. By using the closed-loop model of translational initiation as the basis for our engineering and a genetically encoded poly(A) removal strategy to reduce crosstalk with the endogenous translational machinery, we have defined a new accession point for transgene control in mammalian cells (Fig. 1). This not only expands the cell engineering toolbox for synthetic biology research, but also highlights the importance of a genetically encodable and closed-loop compatible regulation scheme in the quest for effective translational control systems in mammalian cells and eventually in animals. Though mRNA transcripts completely lacking an intact poly(A) tail or any other synthetic eIF4F-interacting moiety in the 3′-UTR can still be translated to produce functional proteins, the absolute expression levels in a maximally activated state in such cases all appear in a similarly low range as we have observed for various cap-independent translational regulation strategies (Supplementary information, Fig. S2f, g). For therapeutic applications in vivo in particular, it is often more important to allow absolute expression levels of the therapeutic protein to reach a specific efficacy window rather than to merely seek for the highest induction folds. For regulation of insulin expression for example, there is a higher priority to ensure that a gene switch produces sufficient amounts of insulin in its activated state (i.e., > 0.4 µg/L, ref. ^3^) rather than being “too tight” so that basal and activated states might both fail to elicit therapeutic responses (Supplementary information, Fig. S7d). When regulating Bax expression, we showed that pro-apoptotic effects were only triggered when transcript levels exceeded cycle threshhold (C_T_) value of 20 (Supplementary information, Fig. S10c), indicating that a corresponding therapeutic gene switch might even tolerate basal Bax levels of such relatively high ranges. Nevertheless, our data support the idea that closed-loop compatible designs, such as strategies capitalizing on regulated inhibition of mRNA self-cleavage^20^ or the STIF-mediated regulation framework described in this work, are generally advantageous for achieving high dynamic ranges (Fig. 1e). Thus, our results indicate that, although the formation of the closed-loop configuration might not be the most critical feature prohibiting functional protein translation in mammalian cells, it strikingly enhances the efficiency of eukaryotic translational inhibition in both natural and synthetic biological contexts.

A broadly and easily adjustable design framework is another key aspect of an effective and versatile translational regulation system with high therapeutic utility. Among other applications, gene switches can be used to experimentally study specific cellular events with high spatiotemporal precision, to monitor critical bioprocess activities during industrial production, or to remotely control therapeutic transgene expression in gene- and cell-based therapies. At the same time, genetically encoded sensors enable cells to detect and respond to critical biological states that may hardly be accessible to conventional diagnostic tools. In this regard, the modular nature of the chimeric STIF-design architecture is highly advantageous for the engineering of genetic switches and sensors responding to any trigger signal of interest as long as suitable sets of proteinaceous regulatory tools are available (Fig. 2). In contrast, though regulation strategies based on ligand-dependent ribozyme inhibition can show excellent performance both in mammalian cells^20^ and in vivo,^48^ one critical limitation of this approach is the poor generalizability of rendering gene expression dependent on any kind of medically relevant target metabolite(s). In fact, the inaugural implementation of this system using bacteriophage λ N-peptide to modulate ribozyme activity^20^ essentially relied on the surprising structural similarity between the N-peptide-specific boxB-aptamer and a unique stem-loop HHR motif, which allowed insertion of boxB into the HHR framework to yield functional aptamer–ribozyme (aptazyme) hybrids. However, a major drawback of this approach is that it not only requires a case-by-case selection of functional aptamers for each ligand, but also non-intuitive redesign of intricate aptazyme structures for each application. Furthermore, this system is incompatible with protein–protein interaction-based split-complementation approaches (Fig. 2b), which makes flexible adjustment of ligand sensing difficult. Nonetheless, despite these issues, this strategy was elegantly repurposed to sense RNA molecules as a type of input signal, allowing ribozyme-specific antisense oligonucleotides to disrupt the catalytically active conformation and activate translation.^48^ Therefore, this system might have potential for the engineering of nucleic acid sensors for various applications.^49^ In contrast, our STIF-based design framework can be easily and flexibly adjusted to create gene switches and sensors responding to many types of control signals, such as chemicals (Fig. 2c), light (Fig. 2c; Supplementary information, Fig. S4b), proteins (Fig. 2e) and signaling dynamics (Fig. 2d). Our results suggest that, as a general design principle, effective transgene control requires a careful balancing act among (i) the affinity of the RBP:aptamer interaction controlling cognate mRNA binding, (ii) the affinity of the interaction between the eIFBP (e.g., NSP3) and the eIF4F complex mediating *trans*-activation and (iii) the regulatory dynamics of the protein–protein interaction system determining the ultimate performance of trigger-inducible gene expression. Overall, we anticipate that the conditional protein association module is the most critical part for the engineering of functional gene switches and sensors; protein–protein interactions reaching an estimated affinity threshold of at least 10 nM are expected to significantly activate STIF-dependent translational initiation.

In line with this expectation, we were able to engineer a grazoprevir-inducible gene switch based on the high-affinity grazoprevir:NS3a interaction (*K*_i_ = 140 pM^33^), which provided rapid, tightly controlled activation kinetics in vitro, as well as good compatibility with therapeutic transgene delivery in vivo (Fig. 3). At present, many synthetic biology-inspired transgene regulation strategies primarily focus on cell therapy applications, including a multitude of “next-generation” CAR-T therapies for cancer^50^ or designer cell-based therapies to treat various metabolic diseases.^51^ Here, we show that STIF-dependent gene switches and sensors are also compatible with state-of-the-art gene therapy delivery strategies. In fact, the translation-based regulatory components could be delivered into living tissues using DNA-encoded vectors or directly formulated as RNA therapeutics, potentially setting the stage for synthetic biology-inspired programmable gene therapies offering marked advantages over counterpart cell therapy concepts in terms of manufacturability and clinical eligibility.

Perhaps the greatest benefit of translation-based transgene regulation is the capability of engineering genetically encoded sensors for multiple intracellular targets of interest (Fig. 4). Although many genetically encoded fluorescent sensors have also been developed on the basis of protein–protein interaction-dependent reconstitution of split fluorescent proteins,^52^ fluorescence-based sensors are limited to visualization-based applications. The programming of more customized sense-and-response activities, such as therapeutic activity, could be more readily achieved with genetic sensors that directly couple constant detection of target compounds to the initiation of expression of target genes of interest. Indeed, such sensors have been developed, for example, allowing cancer-specific promoter activities to initiate therapeutic gene expression.^53^ However, transcription-based systems are inherently limited in that they can only detect target molecules residing in or translocating into the nucleus, which substantially restricts the variety of possible mammalian cell-based applications. Translation-based systems, on the contrary, can detect cytosolic proteins in mammalian cells.^9,20^ For example, one approach results from meticulous engineering work carried out over the past 3 years^9,25^ and capitalizes on caliciviral eIF4E-binding VPg protein, which initiates translation of synthetic mRNA containing chemically modified 5′-UTRs allowing for RBP-mediated VPg recruitment.^9^ Similar to our STIF-based design framework, this strategy takes advantage of a modular split-complementation approach to achieve broadly adjustable transgene control.^9,25^ However, this strategy is only compatible with synthetic mRNAs that contain specific chemical modifications and might therefore be limited to applications such as short-lived RNA drugs or various in vitro cell purification procedures.^54^ So far, this approach remains inapplicable for either genetically encoded expression over longer time periods or integration into complex multi-layered gene circuits to achieve programmable activity in vivo. In contrast, STIF-dependent genetic sensors not only enable generalizable detection of intracellular target signals with high spatial resolution (Fig. 4), but also allow for genetically encoded expression in various physiological contexts (Fig. 5) with seamless integration into complex biocomputational gene circuits for self-sufficient and highly programmable therapies (Fig. 6). Notably, the closed-loop design strategy is particularly suitable for early detection of gene fusions, such as the representative BCR-ABL oncoprotein for CML.^44^ Because gene fusions often result from chromosomal translocations that randomly join two naturally unrelated proteins that do not interact with each other under normal conditions (Supplementary information, Fig. S9a), bi-partite STIF systems where each regulatory part contains a specific domain that binds each individual protein in its native form can only be activated in cancer cells where both domains are abnormally linked within the same molecule (Supplementary information, Fig. S9c). Indeed, our results that simultaneous overexpression of both BCR and ABL genes cannot activate the fusion gene sensor confirm the expected selectivity for the oncoprotein variant (Supplementary information, Fig. S9b). In the future, development of various fusion gene sensors for clinical oncology can follow such design blueprint and kick off a new class of in vivo gene therapies treating various cancers in a similarly self-sufficient fashion as proposed in Fig. 5.

In summary, this work describes a versatile regulation framework based on a new accession point to control translational initiation. Regulation of therapeutic transgenes can be engineered to depend on a multitude of biomedically relevant trigger signals, enabling disease-specific customization of various programmable gene therapies in vivo. This work also adds protein sensing to the portfolio of cell-state classification strategies and describes a class of protein-responsive gene therapies for disease treatments in vivo. Specifically, we anticipate that such protein-driven therapeutic programs could particularly offer benefits for treating various forms of cancer and infectious diseases where a pathologic cell state is unambigously identified by the presence of a specific target protein in the cytosol. For diseases with more complex cellular signatures, we show that STIF-enabled sensors can also be flexibly interconnected with other currently established cell-state classification strategies to create higher-order “therapeutic biocomputers” capable of initiating custom treatment programs according to any desired combination of tissue- and target-specificity in vivo (Fig. 6). Evidently, the STIF architecture is not limited to protein sensing, but is amenable to the detection of any intracellular target of interest (Fig. 2) for which suitable sets of binder moieties (e.g., nanobodies or aptamers) can be found. Thus, the goal of treating various complex diseases through “designable medicines” created through synthetic biology-inspired bioengineering could soon become a clinical reality.

## Materials and methods

### Vector design

References and construction details for all expression vectors are provided in Supplementary information, Table S2. Some expression vectors were constructed by in-fusion cloning using the Seamless Cloning Kit (Beyotime Biotechnology, Shanghai, China; Cat# D7010M). PCR-amplification reactions were performed using KOD One PCR Master Mix (Toyobo Inc., Osaka, Japan; Cat# KMM-201). Ligation reactions were performed using T4 DNA ligase (New England Biolabs, Beverly, MA, USA; Cat# M0202L). Restriction endonucleases were purchased from New England Biolabs. Unpublished plasmids pAdDeltaF6 (Addgene plasmid #112867) and pAAV2/8 (Addgene plasmid #112864) were gifts from James M. Wilson (University of Pennsylvania). pSG5-lamda4G was kindly provided by Matthias W. Hentze.^55^

### Chemicals and recombinant proteins

Abscisic acid (Cat# A8060; stock solution 100 mM in DMSO) and dimethyl sulfoxide (DMSO; Cat# D8371) were purchased from Solarbio Life Sciences (Beijing, China). Danoprevir (Cat# HY-10238; stock solution 10 mM in DMSO) and gibberellic acid (Cat# HY-N1964; stock solution 100 mM in DMSO) were purchased from MedChemExpress (Monmouth Junction, NJ, USA). Animal-free recombinant human EGF (Cat# AF-100-15; 1000× stock solution in ddH_2_O) was purchased from PeproTech EC Ltd. (London, United Kingdom). Grazoprevir (Cat# S3728; stock solution 10 mM in DMSO (for in vitro experiments) or 100 g/L in DMSO (for in vivo experiments)) was purchased from Selleck Chemicals (Houston, TX, USA). Rapamycin (Cat# MC0181; stock solution 10 mM in DMSO) was purchased from ImmunoWay Biotechnology (Plano, TX, USA). Rapamycin analogue (Rapalog; Cat# 535057; stock solution 100 μM in ethanol (EtOH)) was purchased from Takara Bio Inc. (Kusatsu, Japan). 4-Nitrophenyl phosphate disodium salt hexahydrate (pNPP; Cat# 333338-18-4) was purchased from Aladdin Biochemical Technology (Shanghai, China). Phenylmethylsulfonyl fluoride (PMSF, 100 mM solution; Cat# ST506-2) and poly-D-lysine (5 mg/mL solution; Cat# C0312) were purchased from Beyotime Biotechnology (Shanghai, China). Diethanolamine (DEA; Cat# D807525) and EtOH anhydrous (Cat# E809056) were purchased from Macklin Inc. (Shanghai, China). Polyethyleneimine MAX (PEI; Cat# 24765; stock solution 1 mg/mL in ddH_2_O) was purchased from Polysciences (Eppelheim, Germany). Isopropanol (Cat# R018247) was purchased from Rhawn Chemicals (Shanghai, China). D-glucose anhydrous (dextrose; Cat# A610219), glycerol (Cat# A501745; stock solution 10% w/w in ddH_2_O), L-homoarginine hydrochloride (Cat# A602842), magnesium chloride hexahydrate (MgCl_2_; Cat# A610328) and Tween-20 (Cat# A100777) were purchased from Sangon Biotech (Shanghai, China). 4% paraformaldehyde (PFA) solution (Cat# R20497) was purchased from Shanghai Yuanye Bio-Technology (Shanghai, China). Streptozotocin (STZ; Cat# s0130) was purchased from Sigma-Aldrich (MilliporeSigma; Burlington, MA, USA). Calcium chloride anhydrous (CaCl_2_; Cat# 10005861), chloroform (Cat# 10006818), sodium chloride (NaCl; Cat# 10019318; stock solution 5 M in ddH_2_O), potassium chloride (KCl; Cat# 10016308), sodium acetate (Cat# 10018818; stock solution 3 M in ddH_2_O) and trisodium citrate dihydrate (Cat# 10019418) were purchased from Sinopharm Chemical Reagent (Shanghai, China). Puromycin dihydrochloride (Cat# A1113803) and Blasticidin S HCl (Cat# R21001) were purchased from Thermo Fisher Scientific (Waltham, MA, USA). Triton X-100 (Cat# X11206) was purchased from Xinyu Biological Technology (Shanghai, China). Murine RNase inhibitor (Cat# R301) was purchased from Vazyme Biotech (Nanjing, China). Home-made stock solutions of 1 M Tris-HCl (pH 7.5) and 0.5 M EDTA were provided by Westlake University Core Facility.

### Cell culture and transfection

Cell lines derived from human embryonic kidney cells (HEK-293T; ATCC CRL-3216), murine hepatoma cells (Hepa1-6; ATCC CRL-1830) and murine neuroblasts (N2A; ATCC CRL-131) were cultivated in Dulbecco’s modified Eagle’s medium (DMEM; Thermo Fisher Scientific, Waltham, MA, USA; Cat# 12100046) supplemented with 10% (v/v) FBS (Gibco, Australia; Thermo Fisher Scientific, Waltham, MA, USA; Cat# 10099141, lot# 2177370) and 1% (v/v) penicillin/streptomycin solution (PenStrep; Beyotime Biotechnology, Shanghai, China; Cat# ST488). Cell line derived from murine melanoma (B16-F10; ATCC CRL-6475) was cultivated in Roswell Park Memorial Institute 1640 Medium (RPMI 1640; Sartorius AG, Göttingen, Germany; Cat# 01-100-1A) supplemented with 10% (v/v) FBS and 1% PenStrep. All cells were cultured at 37 °C in a humidified atmosphere of 5% CO_2_ in air. For passaging, cells of pre-confluent cultures were detached by incubation in 0.05% trypsin-EDTA (Sangon Biotech, Shanghai, China; Cat# A610629-0050; lot# F319BA0030) for 3 min at 37 °C, collected in 10 mL of cell culture medium, centrifuged for 2 min at 1000 rpm, and resuspended in fresh culture medium at 1.5 × 10^5^ cells/mL, and then seeded into new tissue culture plates. Cell number and viability were quantified using an Invitrogen Countess II AMQAX1000 Cell Counter (Thermo Fisher Scientific; Cat# AMQAX1000).

Unless indicated otherwise, transfection was performed at 12 h after seeding 5 × 10^4^ mammalian cells into each well of a 24-well plate. The cell culture medium was replaced with fresh medium (not containing transfection reagents) at 6 h after transfection. HEK-293T cells were transfected using a PEI-based protocol at a PEI:DNA ratio of 5:1 (w/w) and in a transfection volume of 50 µL native serum-free DMEM per well. N2A, Hepa1-6 and B16-F10 cells were transfected using Lipofectamine 3000 (Thermo Fisher Scientific, Cat# L3000015) reagent according to the manufacturer’s instructions. In vitro-transcribed mRNA was transfected by mixing 0.75 µL Lipofectamine 3000 (without the P3000 reagent) with 500 ng nucleic acids in a transfection volume of 50 µL native serum-free Opti-MEM medium (Thermo Fisher Scientific, Cat# 31985062) per well. Transfection agents and nucleic acids were incubated for 15 min at 25 °C before dropwise addition to cells. For RNA transfection, cell culture medium was replaced with Lipofectamine 3000-free medium at 4 h post transfection.

### Generation of stable cell lines

Polyclonal HEK_EGFP-NS3a(H1)_, N2A_EGFP-NS3a(H1)_, Hepa1-6_EGFP-NS3a(H1)_, and B16-F10_EGFP-NS3a(H1)_ populations, transgenic for stable expression of EGFP-NS3a(H1), were constructed by co-transfecting 500 ng pSL816 and 5 ng pCMV-T7-SB100 into 5 × 10^4^ native HEK-293T, N2A, Hepa1-6, and B16-F10 cells, respectively. After selection with 100 µg/mL puromycin dihydrochloride, 10% of the surviving population with the highest EGFP expression was subjected to fluorescence-activated cell sorting using the MA900 Multi-Application Cell Sorter (Sony Biotechnology; San Jose, CA, USA). The polyclonal HEK_SEAP_ cell line was constructed by co-transfecting 500 ng pSL1314 and 5 ng pCMV-T7-SB100 into 5 × 10^4^ native HEK-293T cells and selected with 10 µg/mL Blasticidin S HCl for constitutively high SEAP expression.

### In vitro transcription

Template DNA fragments containing a T7 promoter were isolated from corresponding plasmids by restriction endonuclease treatment and transcribed with a T7 High Yield RNA Transcription Kit (Vazyme Biotech, Nanjing, China; Cat# TR-101) after the addition of 40 mM 3′-O-Me-m^7^G(5′)ppp(5′)G RNA Cap Structure Analog (New England Biolabs, Beverly, MA, USA; Cat# S1411L). After the removal of template DNA using 1 U RNase-free DNaseI (Vazyme Biotech; Cat# EN401-01), mRNA was purified by precipitation with 0.3 M sodium acetate at –20 °C, followed by centrifugation at 4 °C and 15,000 rpm for 30 min and washing with 70% EtOH. Purified mRNA was re-suspended in RNase-free ddH_2_O and RNA was quantified by measuring UV absorption with a UVP Crosslinker CL-3000 (Analytik Jena GmbH, Jena, Germany).

### RNA extraction and cDNA synthesis

Total RNA of cells was isolated using the Trizol RNA extraction method. In brief, 1 × 10^6^ cells were mixed with 1 mL TRIzol™ Reagent (Thermo Fisher Scientific, Cat# 15596018) and vortexed until no precipitate could be observed. The cell lysate was then mixed with 200 μL chloroform and centrifuged at 12,000× *g* at 4 °C for 15 min. The aqueous phase was collected, mixed with 500 μL isopropanol and incubated at –20 °C for 30 min to precipitate RNA. The RNA pellet was harvested by centrifugation at 4 °C and 15,000× *g* for 30 min, washed with 70% EtOH, air-dried and resuspended in 50 μL RNase-free water. For cDNA synthesis, 1 µg RNA was taken for cDNA synthesis using the HiScript II Q RT SuperMix (Vazyme Biotech; Cat# R223-01) for qPCR.

### Reverse transcription-PCR (RT-PCR)

For quantitative analysis, PCR reaction was carried out with an initial step of 95 °C for 30 s followed by 40 cycles of 95 °C for 10 s and 60 °C for 30 s on the Applied Biosystems QuantStudio1 Real-Time PCR System (Thermo Fisher Scientific) using the ChamQ Universal SYBR qPCR Master Mix (Vazyme Biotech; Cat# Q711-02) and the primers listed in Supplementary information, Table S1. The relative C_T_ value was determined and normalized against the expression level of endogenous human glyceraldehyde 3-phosphate dehydrogenase (GAPDH) or murine ribosomal protein (Rplp0) coding genes.

### RNA immunoprecipitation-qPCR

At 48 h after transfection, cells were harvested with ice-cold lab-made PBS (Westlake University Core Facility), resuspended in RNase-free NETN300 cell lysis buffer (50 mM Tris-HCl, 300 mM NaCl, 2 mM EDTA, 0.05% Triton X-100, 1 mM PMSF and 50 U/mL murine RNase inhibitor) and incubated for 15 min on ice. Following centrifugation at 16,000× *g* and 4 °C for 15 min, 4% of the supernatant was collected as the “input sample” and the remaining supernatant was diluted with RNase-free NETN0 Buffer (NETN300 cell lysis buffer without NaCl) to reach a final NaCl concentration of 100 mM before samples were immunoprecipitated through incubation with anti-Flag affinity gel (Beyotime Biotechnology; Cat# P2271) for 90 min at 4 °C. The anti-Flag affinity gel was then centrifuged at 6000× *g* for 30 s and washed 3 times with ice-cold NETN3000 wash buffer (50 mM Tris-HCl, 300 mM NaCl, 0.05% Triton X-100 and 1 mM PMSF). RNA in both the “input sample” and affinity gel was extracted for RT-PCR analysis. The method was adapted from that described elsewhere.^56^

### Co-immunoprecipitation

At 48 h after transfection, cells were harvested and lysed for 30 min at 4 °C with cell lysis buffer (Beyotime Biotechnology; Cat# P0013) supplemented with 1 mM PMSF. Following centrifugation at 14,000× *g* for 10 min, 100 µL of supernatant was collected as the “input sample” and the remaining supernatant was immunoprecipitated by incubation for 3 h at 4 °C with anti-Flag affinity gel (Beyotime Biotechnology; Cat# P2271). The anti-Flag affinity gel was then centrifuged at 6000× *g* for 30 s and washed 3 times with IP wash buffer (20 mM Tris-HCl, 0.2 mM EDTA, 100 mM KCl, 2 mM MgCl_2_, 0.1% Tween 20 and 10% glycerol). Proteins in both the “input sample” and affinity gel were mixed with 5× SDS-PAGE loading buffer (Beyotime Biotechnology; Cat# P0015), boiled at 98 °C for 10 min and prepared for western blot. The method was adapted from that described elsewhere.^57^

### Western blot

After treatment with 5× SDS-PAGE loading buffer (Beyotime Biotechnology; Cat# P0015) at 98 °C for 10 min, samples were resolved on 8%, 10% or 12% SDS polyacrylamide gels from the SDS-PAGE preparation kit (Sangon Biotech, Shanghai, China; Cat# C631100) and electroblotted onto polyvinylidene fluoride western blot membranes (Merck Millipore KGaA, Darmstadt, Germany; Cat# 03010040001). Membranes were incubated in BeyoECL Moon detection reagent (Beyotime Biotechnology; Cat# P0018FS) and visualized using the Amersham Imager 600 (AI600 RGB; GE Healthcare, Uppsala, Sweden) after application of primary rabbit anti-Flag antibody (Sigma-Aldrich; Cat# F7425), rabbit anti-HA antibody (Cell Signaling Technology, Danvers, MA, USA; Cat# 3724; lot# 10), rabbit anti-eIF4G antibody (Cell Signaling Technology; Cat# 2498; lot# 4), rabbit anti-eIF4E antibody (Cell Signaling Technology; Cat# 2067; lot# 8), rabbit anti-GAPDH (6C5) antibody (Santa Cruz Biotechnology; Cat# sc-32233; lot# L2019) or rabbit anti-Bax antibody (Cell Signaling Technology; Cat# 14796; lot# 800) and secondary HRP-labeled goat anti-rabbit IgG (Beyotime Biotechnology; Cat# A0208; lot# 110219200406).

### Quantification of target gene expression

Expression levels of human placental SEAP in culture supernatants were quantified based on p-nitrophenyl phosphate-derived light absorbance at 415 nm.^58^ SEAP levels in mouse serum were profiled using a SEAP chemiluminescence assay kit (Roche Diagnostics GmbH, Mannheim, Germany; Cat# 11 779 842 001). NanoLuc levels were profiled using the Nano-Glo^®^ Luciferase Assay System (Promega, Madison, WI, USA; Cat# N1120). Firefly luciferase levels were profiled using the Luciferase Reporter Gene Assay Kit (Yeasen Biotechnology, Shanghai, China; Cat# 11401ES60) after lysis of the cells for 15 min at 4 °C, followed by centrifugation at 12,000× *g* for 5 min. Rodent modified insulin (mINS) levels in culture supernatants and mouse serum were quantified with a mouse insulin ELISA kit (Mercodia Inc., Uppsala, Sweden; Cat# 10-1247-01).

### Flow cytometry

Cell populations were analyzed with a CytoFLEX LX flow cytometer (Beckman Coulter, Indianapolis, IN, USA) equipped for detection of EGFP (488 nm laser, 525/40 emission filter) and mCherry (561 nm laser, 610/20 emission filter) and set to exclude dead cells and cell doublets. 10,000 cells were recorded per data set and analyzed with FlowJo™ software (v10; BD Biosciences). Weighted EGFP or mCherry expression levels were determined by setting an arbitrary threshold of 10^5^ fluorescence units and multiplying the percentage of gated cells by their median fluorescence.

### Fluorescence imaging

Fluorescence microscopy was performed with a Nikon ECLIPSE Ts2-FL fluorescence microscope (Nikon Instruments Inc., Melville, NY, USA) equipped with a C-mount camera, F-mount camera, a 20× objective, an excitation and emission filter set (EGFP: 488/509 nm; mCherry: 587/610 nm) and OPLENIC software (version x64, 10.1.14643.20190511).

### Confocal microscopy

At 24 h after transfecting 1 µg plasmid DNA into 1 × 10^5^ cells seeded on a 20 mm glass-bottomed cell culture dish (Wuxi NEST Biotechnology, Wuxi, China; Cat# 801001) coated with 5 mg/mL poly-D-lysine, the cell culture medium was removed and cells were washed with 1 mL PBS and fixed with 1 mL 4% PFA solution. After 10 min, cells were washed with PBS and stained with DAPI staining solution (Beyotime Biotechnology; Cat# C1005) for 15 min in dark. Finally, the cells were washed three times with PBS and imaged with a A1R HD25 confocal microscope (Nikon Instruments Inc., Melville, NY, USA).

### AAV production

AAV2/8-(GNCR1)_3_-NSP3, AAV2/8-MCP-(NS3a)_3_ and AAV2/8-SEAP-(MS2-box)_24_-HHR-pA were produced by PackGene Biotech (Guangzhou, China) using the transfer plasmids pSL511, pSL512 and pSL446, respectively.

### Animal experiments

Animal experiments were performed according to the protocol (Protocol ID: 20-001-XMQ) approved by the Institutional Animal Care and Use Committee (IACUC) of Westlake University and in accordance with the Animal Care Guidelines of the Ministry of Science and Technology of the People’s Republic of China.

#### Hydrodynamic tail vein injection

Endotoxin-free plasmids were diluted in Ringer’s solution (147 mM NaCl, 4 mM KCl, 1.13 mM CaCl_2_) to reach final injection volume of 100 µL per gram body-weight and injected into tail veins of > 6-week-old mice using 5 mL syringes.

#### STZ T1D mouse model

Fasted 6-week-old male WT C57BL/6 mice were injected daily with freshly diluted STZ (50 mg/kg in 200 µL ice-cold sodium citrate buffer) for five consecutive days. Chronic fasting hyperglycemia (> 15 mM) developed after 3 weeks.

#### Xenograft tumor model

1 × 10^6^ B16-F10 or 2 × 10^6^ Hepa1-6 cells in 0.1 mL sterile PBS were subcutaneously injected into the right lower back of 4-week-old male WT C57BL/6 mice. After 7 days, each animal received intratumoral injection of 60 µL Lipofectamine 3000 solution containing 20 μg of plasmid DNA on different days after cell implantation under anesthesia.

#### Drug administration

Grazoprevir (100 μg/µL) was administered by intraperitoneal injection or oral gavage.

#### Blood sampling

Whole blood was collected from the submandibular vein of mice and clotted by incubation at 4 °C for 2 h, and then serum was isolated by centrifugation for 8 min at 8000× *g*.

#### Glycemia measurement

Glycemia of mice was measured with a commercial glucometer (Sinocare plus Code Glucometer; detection range: 1.1–33.3 mM) purchased from a local pharmacy.

#### GTT

D-glucose was freshly prepared and intraperitoneally injected into mice at a 0.75 g/kg dose before time zero.

### Data analysis

Two-tailed unpaired Student’s *t*-tests were used to evaluate the statistical significance of differences between two groups. For tumor volume studies, two-way ANOVA was used for statistical analysis. *P* values < 0.05 were considered statistically significant. Statistical parameters and corresponding *P* values are included in the figure legends. All analyses were performed using GraphPad Prism 9 (Graph Pad Software, San Diego, CA, USA).

### Supplementary information

Supplementary information, Fig. S1

Supplementary information, Fig. S2

Supplementary information, Fig. S3

Supplementary information, Fig. S4

Supplementary information, Fig. S5

Supplementary information, Fig. S6

Supplementary information, Fig. S7

Supplementary information, Fig. S8

Supplementary information, Fig. S9

Supplementary information, Fig. S10

Supplementary information, Table S1

Supplementary information, Table S2

Supplementary information, Table S3

## Data Availability

The data that support the findings of this study are available upon reasonable request from the lead corresponding author (M.X.).
