# Supplementary material for: Engineered poly(A)-surrogates for translational regulation and therapeutic biocomputation in mammalian cells
Source: Cell Res. 2024 Jan 4;34(1):31–46. doi: 10.1038/s41422-023-00896-y (PMC10770082; doi:10.1038/s41422-023-00896-y)
Supplement: Supplementary file 1 — Supplementary information, Fig. S1 [file 41422_2023_896_MOESM1_ESM.pdf]

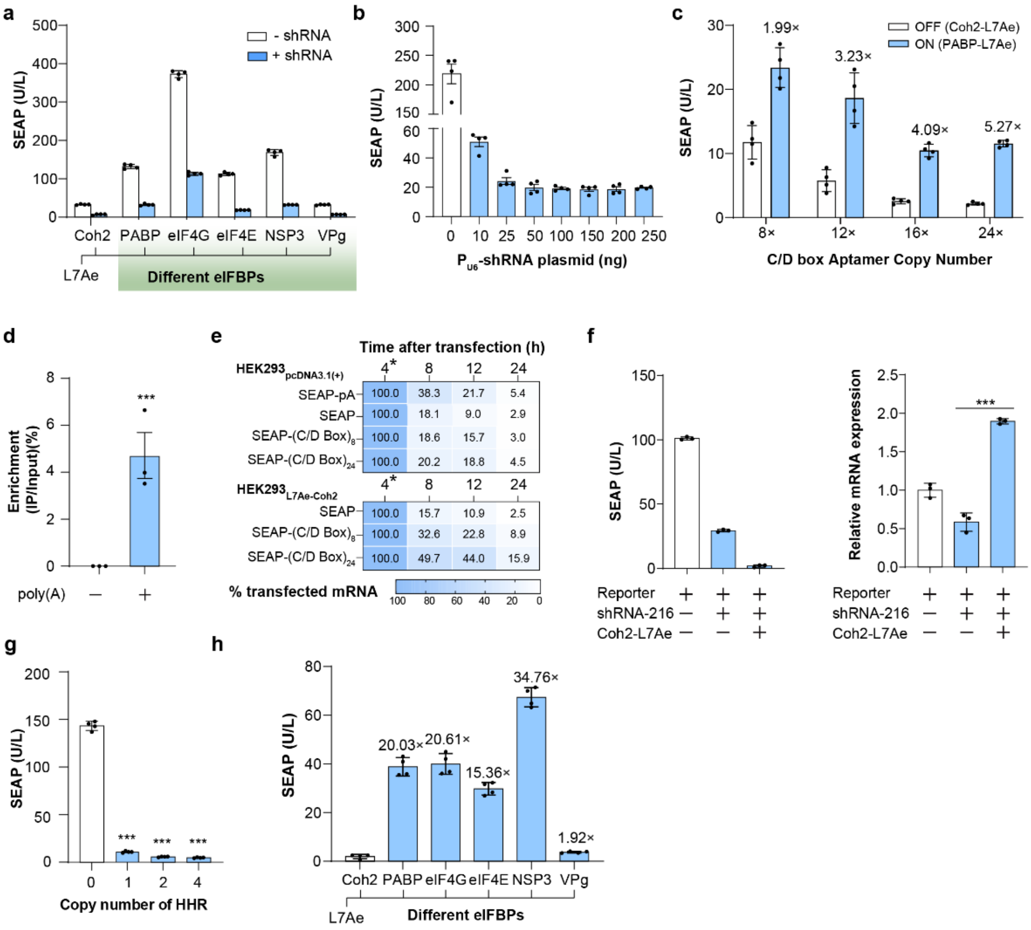

Fig. S1. Control experiments related to Fig. 1. (a) Synthetic translation initiation factors (STIFs)-mediated translation of SEAP mRNA containing shRNA-216-cleavable poly(A). HEK-293 cells were co-transfected with a SEAP expression vector containing 8 tandem L7Ae-specific C/D-box repeats (pSL31), an shRNA-216 expression vector (pSL4, 100ng) and expression vectors for different STIF variants (PABP-L7Ae, pLZ16; eIF4G-L7Ae, pWH127; L7Ae-eIF4E, pDJ55; L7Ae-NSP3, pLZ27; L7Ae-VPg, pLZ248) or an L7Ae-Coh2 protein incapable of translational initiation (pSL44, negative control). SEAP levels in culture supernatants were quantified at 48 h post transfection. Data are shown as the mean ± SD of n = 4 independent experiments. **(b) Validation of mRNA knockdown efficiency of shRNA-216.** HEK-293 cells were co-transfected with an expression vector for shRNA-216-repressible SEAP-mRNA (pSL31) and different amounts of a P_{hU6}-driven shRNA-216 expression vector (pSL4, 0 to 250ng). SEAP expression in the culture supernatant was scored at 48h after transfection. Data presented are mean ± SD of n = 4 individual

experiments. **(c) Correlation between aptamer region size and STIF-mediated SEAP expression.** HEK-293 cells were co-transfected with SEAP expression vectors containing different tandem repeats of the L7Ae-specific C/D-box aptamer ((C/D-box)$_8$, pSL31; (C/D-box)$_{12}$, pSL81; (C/D-box)$_{16}$, pSL80; (C/D-box)$_{24}$, pSL88), an shRNA-216 expression vector (pSL4, 100ng) and an expression vector for either PABP-L7Ae (ON; pLZ16) or a Coh2-L7Ae protein incapable of binding eIF4F (OFF; pSL44). SEAP expression in culture supernatants was scored at 48 h post transfection. Data presented are mean ± SD, n = 4 individual experiments. **(d) Quantification of the binding capability of PABP to poly(A)-containing RNA by RIP-qPCR.** HEK-293 cells were transfected with a constitutive expression vector for 3xFLAG-tagged PABP-L7Ae reflecting the RNA binding capacity of endogenous PABP (P_{hCMV}-PABP-L7Ae-3xFLAG-pA, pSL763). After 24 h, 10 µg of *in vitro*-transcribed EGFP mRNA with (+) or without (-) a poly(A) tail was added. At 3h after transfection, RNA was extracted and co-immunoprecipitated using anti-Flag affinity gel. Data show the results of qRT-PCR analysis as the ratio (%) of EGFP-mRNA in samples before (input) and after immunoprecipitation (IP) (± SD, n = 3). **(e) Impact of engineered L7Ae-specific poly(A) surrogates on mRNA stability.** SEAP-mRNA *in vitro*-transcribed to contain poly(A) (from pSL1091), no poly(A) (from pSL517) or poly(A)-surrogates consisting of 8 (from pSL31) or 24 tandem C/D-box repeats in the 3'-UTR (from pSL355) was transfected into HEK-293 cells expressing Coh2-L7Ae (24 h after transfection of pSL44) or not expressing Coh2-L7Ae (24 h after transfection of pcDNA3.1(+)). SEAP-mRNA levels were analyzed by qRT-PCR at 4 (*arbitrarily set as 100%), 8, 12 and 24 hours after mRNA transfection. Data presented are mean ± SD, n = 4 independent experiments. **(f) mRNA stabilization through ABP/aptamer binding.** HEK-293 cells were co-transfected with a SEAP expression vector (reporter; pSL88) and constitutive expression vectors for shRNA-216 (pSL4, 100ng) and a Coh2-L7Ae protein incapable of translational initiation (pSL44). SEAP levels in culture supernatants (left) and mRNA expression levels in cells (right) were quantified at 48 h post-transfection. Transcript levels of SEAP were normalized to glyceraldehyde 3-phosphate dehydrogenase (GAPDH) expression by setting undetermined values to a maximum Ct

of 40 cycles. Data presented are mean ± SD, n = 3. **(g) Quantification of HHR-mediated mRNA self-cleavage.** HEK-293 cells were co-transfected with SEAP expression vectors containing zero (pSL517), one (pSLM97), two (pSL767) or four HHR tandem repeats (pSL768) before SEAP levels in culture supernatants were scored at 48h post transfection. Data presented are mean ± SD of n = 4 individual experiments. **(h) STIF-mediated translation of SEAP mRNA containing HHR-cleavable poly(A).** HEK-293 cells were co-transfected with a SEAP expression vector containing 24 tandem L7Ae-specific C/D-box repeats (pSL355) and expression vectors for different STIF variants (PABP-L7Ae, pLZ16; eIF4G-L7Ae, pWH127; L7Ae-eIF4E, pDJ55; L7Ae-NSP3, pLZ27; L7Ae-VPg, pLZ248) or an L7Ae-Coh2 protein incapable of translational initiation (pSL44, negative control). SEAP levels in culture supernatants were quantified at 48 h post transfection. Data are shown as the mean ± SD of n = 4 independent experiments.
