# Supplementary material for: Engineered poly(A)-surrogates for translational regulation and therapeutic biocomputation in mammalian cells
Source: Cell Res. 2024 Jan 4;34(1):31–46. doi: 10.1038/s41422-023-00896-y (PMC10770082; doi:10.1038/s41422-023-00896-y)
Supplement: Supplementary file 2 — Supplementary information, Fig. S2 [file 41422_2023_896_MOESM2_ESM.pdf]

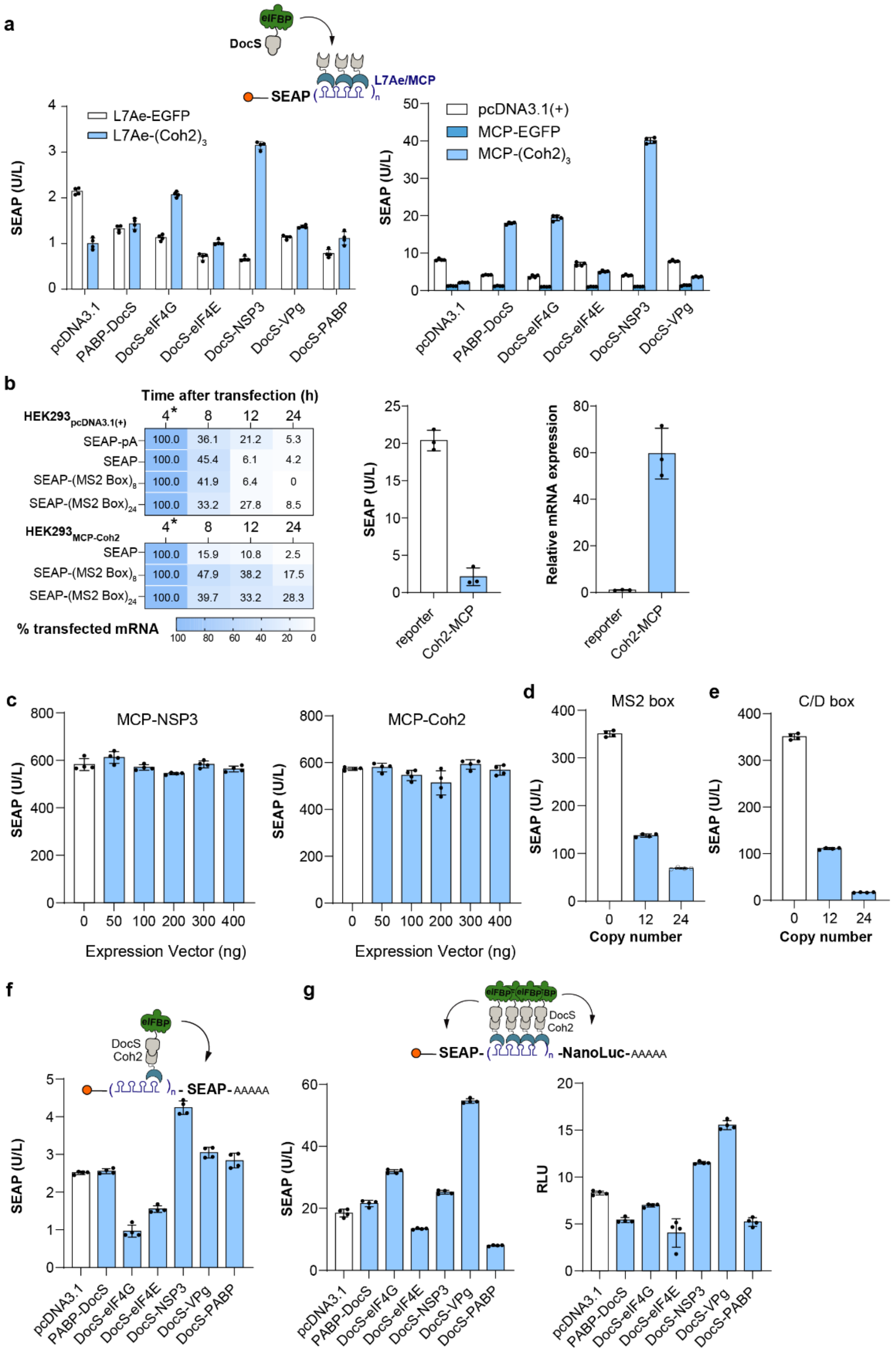

Fig. S2. Translational regulation by spontaneous STIF reconstitution. (a) Site-specific translational activation by eIFBP overexpression. HEK-293 cells were (co-)transfected with an expression vector for SEAP-mRNA containing 24 tandem C/D-box- (left panel, pSL355) or MS2-box repeats in the 3'-UTR (right panel, pSL468),

constitutive expression vectors for L7Ae (left) or MCP (right) fused to Coh2 (L7Ae-Coh2, pSL83; MCP-Coh2, pSL674) or EGFP (L7Ae-EGFP, pSL1078; MCP-EGFP, pSL435) and constitutive expression vectors for various chimeric Coh2-specific DocS-containing eIFBP fusions (PABP-DocS, pSL47; DocS-eIF4G, pSL87; DocS-eIF4E, pLZ312; DocS-NSP3, pSL66; DocS-VPg, pLZ311). Transfection of pcDNA3.1(+) instead of DocS- or MCP-expressing vectors was used as a negative control. SEAP levels in culture supernatants were quantified at 48 h post-transfection. Data are shown as the mean ± SD, n = 4 independent experiments. **(b) Impact of engineered MCP-specific poly(A) surrogates on mRNA stability.** (Left) SEAP-mRNA *in vitro*-transcribed to contain poly(A) (from pSL1091), no poly(A) (from pSL517), or poly(A)-surrogates consisting of 8 (from pSL515) or 24 tandem MS2-box repeats in the 3'-UTR (from pSL468) were transfected into HEK-293 cells expressing MCP-Coh2 (24h after transfection of pSL674) or not expressing MCP-Coh2 (24h after transfection of pcDNA3.1(+)). SEAP-mRNA levels were analyzed with RT-PCR at 4 (*arbitrarily set as 100%), 8, 12, and 24 hours after mRNA transfection. Data presented are mean ± SD, n = 4 independent experiments. (Right) HEK-293 cells were co-transfected with a SEAP expression vector containing 24 MS2-box repeats in the 3'-UTR (reporter; pSL468) and an MCP-Coh2 protein incapable of translational initiation. SEAP levels in culture supernatants and relative mRNA expression levels in cells were quantified at 48 h post-transfection. Transcript levels of SEAP were normalized to glyceraldehyde 3-phosphate dehydrogenase (GAPDH) expression by setting undetermined values to a maximum Ct of 40 cycles. Data presented are mean ± SD, n = 3. **(c) Impact of STIF overexpression on endogenous gene expression.** HEK-293 cells stably transgenic for constitutive SEAP expression (HEK_{SEAP}) were transfected with different amounts of a constitutive expression vector for MCP-NSP3 (left, pSL95) or MCP-Coh2 (right, pSL674). Total amounts of transfected DNA were kept constant using pWS164 (P_{hCMV}-EGFP-pA) as a filler plasmid. SEAP levels in culture supernatants were quantified at 48 h post-transfection. Data are shown as the mean ± SD, n = 4 independent experiments. **(d, e) Impact of poly(A) surrogate size on translation efficiency of reporter mRNA.** HEK-293 cells were transfected with SEAP expression vectors

containing 12 or 24 tandem repeats of MS2-box (D: P_{hCMV}-SEAP-(MS2-box)$_{12}$-pA, pSL1310; P_{hCMV}-SEAP-(MS2 box)$_{24}$-pA, pSL89) or C/D-box aptamers in the 3'-UTR (E: P_{hCMV}-SEAP-(C/D-box)$_{12}$-pA, pSL81; P_{hCMV}-SEAP-(C/D-box)$_{24}$-pA, pSL88). Transfection of a constitutive SEAP expression vector (P_{hCMV}-SEAP-pA, pSL517) was used as positive control representative for 0 aptamer repeats. SEAP expression in culture supernatants was scored at 48 h post-transfection. Data presented are mean ± SD, n = 4 independent experiments. **(f, g) Natural 5'-cap-independent translation by site-specific eIFBP recruitment to the (f) 5'-UTR or (g) intergenic regions of target gene mRNA.** (f) HEK-293 cells were (co-)transfected with an expression vector for SEAP mRNA containing 4 tandem C/D-box repeats in the 5'-UTR (P_{hCMV}-(C/D-box)$_4$-SEAP-pA, pQZ8), a constitutive L7Ae-(Coh2)$_3$ expression vector (pSL83) and constitutive expression vectors for various DocS-based fusion constructs (PABP-DocS, pSL47; DocS-eIF4G, pSL87; DocS-eIF4E, pLZ312; DocS-NSP3, pSL66; DocS-VPg, pLZ311). Transfection of pcDNA3.1(+) instead of DocS-expressing vectors was used as a negative control. SEAP levels in culture supernatants were quantified at 48 h post-transfection. Data are shown as the mean ± SD, n = 4 independent experiments. (g) HEK-293 cells were (co-)transfected with reporter mRNA containing 24 tandem C/D-box repeats placed downstream of SEAP- and upstream of NanoLuc-coding regions (P_{hCMV}-SEAP-(C/D-box)$_{24}$-NanoLuc-pA, pPW21), a constitutive L7Ae-(Coh2)$_3$ expression vector (pSL83) and constitutive expression vectors for various DocS-based fusion constructs (PABP-DocS, pSL47; DocS-eIF4G, pSL87; DocS-eIF4E, pLZ312; DocS-NSP3, pSL66; DocS-VPg, pLZ311). Transfection of pcDNA3.1(+) instead of DocS-expressing vectors was used as a negative control. SEAP (left) and NanoLuc levels in culture supernatants (right) were quantified at 48 h post-transfection. Data are shown as the mean ± SD, n = 4 independent experiments.
