# Supplementary material for: Engineered poly(A)-surrogates for translational regulation and therapeutic biocomputation in mammalian cells
Source: Cell Res. 2024 Jan 4;34(1):31–46. doi: 10.1038/s41422-023-00896-y (PMC10770082; doi:10.1038/s41422-023-00896-y)
Supplement: Supplementary file 3 — Supplementary information, Fig. S3 [file 41422_2023_896_MOESM3_ESM.pdf]

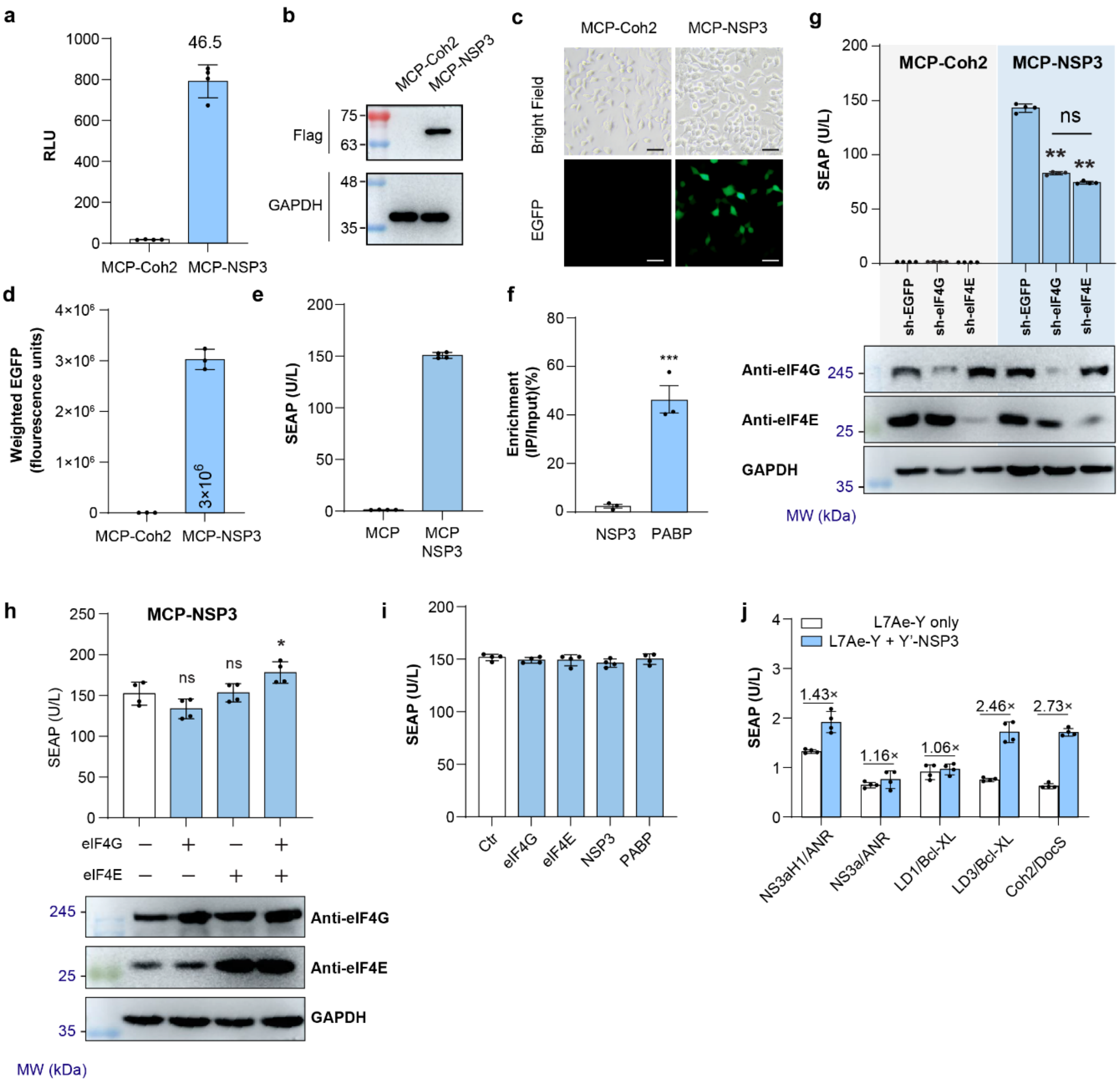

Fig. S3. Control experiments related to Figures 1 and 2. (a, b) STIF-mediated regulation at the protein level of gene expression. HEK-293 cells were co-transfected with an expression vector for FLAG-tagged Firefly Luciferase (FLuc) containing MCP-specific poly(A) surrogates in the 3'-UTR (pSL781) and constitutive expression vectors for MCP-Coh2 (pSL674) or MCP-NSP3 (pSL95). At 48h post-transfection, (a) Luciferase activity and (b) absolute protein levels of FLuc were detected using Western Blot. Data in (a) are shown as the mean ± SD, n = 4 independent experiments. **(c, d) STIF-mediated translational regulation of EGFP-mRNA.** HEK-293 cells were co-transfected with an expression vector for EGFP-mRNA containing 24 tandem MS2-box repeats in the 3'-UTR (pSL1308) and constitutive expression vectors for MCP-

Coh2 (pSL674) or MCP-NSP3 (pSL95). At 48h post-transfection, EGFP expression was scored by (c) fluorescence microscopy (scale bar: 10 µm) or (d) flow cytometry. Data are mean ±SD, n=3. **(e) SEAP expression levels of experiment described in Fig. 1F.** HEK-293 cells were transfected with a SEAP expression vector containing 24 tandem MCP-specific MS2-box repeats (pSL468) and expression vectors for either 3xFLAG-tagged MCP (pSL1084) or 3xFLAG-tagged MCP-NSP3 (pSL1083). Cell lysate and culture medium were harvested at 48h post transfection for immunoprecipitation (Fig. 1F) and SEAP evaluation, respectively. Bars represent the mean SD, n = 4 independent experiments. **(f) Quantification of endogenous RNA binding capacity of PABP- and NSP3-fusion proteins by RIP-qPCR.** At 48 h after transfection of HEK-293 cells with expression vectors for 3xFLAG-tagged L7Ae-NSP3 (pSL762) or PABP-L7Ae (pSL763), RNA was extracted and co-immunoprecipitated using anti-Flag affinity gel. Data show the results of qRT-PCR analysis as the ratio (%) of GAPDH RNA in samples before (input) and after immunoprecipitation (IP), taken as a measure of the amount of RNA bound on PABP- and NSP3-containing constructs (± SD, n = 3). Bars represent the mean and SD, and filled circles show individual results. **(g) Impact of shRNA-mediated eIF4F knockdown on STIF-specific translation.** HEK-293 cells were transfected with a SEAP expression vector containing 24 tandem MCP-specific MS2-box repeats (pSL468), expression vectors for either MCP-Coh2 (pSL674; left) or MCP-NSP3 (pSL95; right), and constitutive expression vectors for shRNAs specific to eIF4G (pSL1431; shR-eIF4G), eIF4E (pSL1432; shR-eIF4E) or EGFP as shRNA-control (pSL260; shR-EGFP). Western Blot analysis of endogenous eIF4G and eIF4E levels (lower panel) and quantification of SEAP levels (upper panel) were performed at 48 h post transfection. Bars represent the mean SD, n = 4 independent experiments. **(h, i) Impact of eIF4F overexpression on STIF-specific translation.** **(h)** HEK-293 cells were transfected with a SEAP expression vector containing 24 tandem MCP-specific MS2-box repeats (pSL468), a constitutive MCP-NSP3 expression vector (pSL95), and different combinations of expression vectors for native eIF4G (pSL1429) and eIF4E (pSL1430). For (-) conditions, pcDNA3.1(+) was transfected instead of eIF4G/eIF4E

expression vectors. Western Blot analysis of endogenous eIF4G and eIF4E levels (lower panel) and quantification of SEAP levels (upper panel) were performed at 48 h post transfection. **(i)** HEK-293 cells were transfected with a SEAP expression vector containing 24 tandem MCP-specific MS2-box repeats (pSL468), a constitutive MCP-NSP3 expression vector (pSL95) and a constitutive expression vector for either eIF4G (pSL1429), eIF4E (pSL1430), NSP3 (pSL1471) or PABP (pSL1470). Transfection of pcDNA3.1(+) instead of eIFBP expression vectors was used as negative control. SEAP levels were scored at 48 h post transfection. Bars represent the mean SD, n = 4 independent experiments. **(j) Translational regulation through constitutive STIF reconstitution.** HEK-293 cells were co-transfected with plasmids encoding SEAP mRNA containing an L7Ae-specific poly(A)-surrogate (pSL355) and constitutive expression vectors for different combinations of L7Ae- and NSP3-fusion proteins (L7Ae-NS3a(H1)&ANR_4-NSP3: pSL703/pSL549; L7Ae-NS3a&ANR_4-NSP3: pYF5/pSL549; L7Ae-LD1&Bcl-XL-NSP3: pSL667/pSL615; L7Ae-LD3&Bcl-XL-NSP3: pSL661/pSL615; L7Ae-Coh2&DocS-NSP3: pSL65/pSL66). Transfection of pcDNA3.1(+) instead of NSP3-fusion proteins was used as a negative control. SEAP levels in culture supernatants were quantified at 48 h post-transfection. Data are shown as the mean ± SD of n = 4 independent experiments. ** $P<0.01$, *** $p<0.001$, by two-tailed unpaired t-test.
