# Supplementary material for: Engineered poly(A)-surrogates for translational regulation and therapeutic biocomputation in mammalian cells
Source: Cell Res. 2024 Jan 4;34(1):31–46. doi: 10.1038/s41422-023-00896-y (PMC10770082; doi:10.1038/s41422-023-00896-y)
Supplement: Supplementary file 4 — Supplementary information, Fig. S4 [file 41422_2023_896_MOESM4_ESM.pdf]

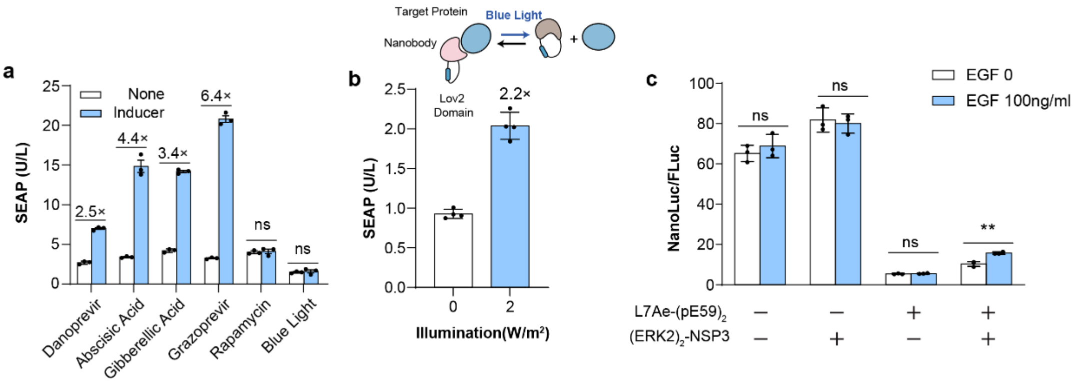

Fig. S4. Control experiments for STIF-based gene switches and intracellular sensors. (a) Translational regulation through trigger-inducible STIF reconstitution. For danoprevir-inducible SEAP translation, HEK-293 cells were co-transfected with pSL355 and constitutive expression vectors for L7Ae-(NS3a)$_3$ (pLZ76) and (DNCR2)$_3$-NSP3 (pLZ72). For abscisic acid-inducible SEAP translation, HEK-293 cells were co-transfected with pSL355 and constitutive expression vectors for L7Ae-(ABI)$_3$ (pPW3) and (PYL)$_3$-NSP3 (pPW4). For gibberellic acid-inducible SEAP translation, HEK-293 cells were co-transfected with pSL355 and constitutive expression vectors for GAI-L7Ae (pPW14) and NSP3-GID (pPW17). For grazoprevir-inducible SEAP translation, HEK-293 cells were co-transfected with pSL355 and constitutive expression vectors for L7Ae-(NS3a)$_3$ (pLZ76) and (GNCR)$_3$-NSP3 (pLZ74). For rapamycin-inducible SEAP translation, HEK-293 cells were co-transfected with pSL355 and constitutive expression vectors for FKBP-L7Ae (pMX331) and FRB-NSP3 (pLZ42). For blue light-inducible SEAP translation, HEK-293 cells were co-transfected with pSL355 and constitutive expression vectors for L7Ae-CIB1 (pLZ55) and Cry2-NSP3 (pLZ68). SEAP levels in culture supernatants were scored at 48 h after addition of corresponding inducers (danoprevir, 1 μM; abscisic acid, 100 μM; gibberellic acid, 100 μM; grazoprevir, 0.5 μM; rapamycin, 0.01μM) or at 24 h after exposure to blue light (450 nm; ON, 30 s at 5 mW/cm^2; OFF, 30 s). Data are shown as the mean ± SD, n = 3. **(b) Light-inducible translational regulation by a blue light-activated mCherry nanobody LaM8-AK47.** HEK-293 cells were transfected with an expression vector for SEAP-mRNA containing 16 tandem MS2-box repeats in the 3’-

UTR (P_{hCMV}-SEAP-(MS2-box)$_{16}$-pA, pSL516) and constitutive expression vectors for MCP-LaM8_AK47 (pSL875) and mCherry-NSP3 (pSL876). At 6h post transfection, cells were illuminated for 48h with blue light (450 nm, 2 mW/cm^2) before SEAP levels in culture supernatants were scored. Data are mean±SD, n=4. **(c) Genetically encoded signalling-specific sensors employing phosphorylation-dependent STIF reconstitution (L7Ae-version of Fig. 2d).** HEK-293 cells were co-transfected with a dual reporter vector containing a constitutive FLuc expression unit and an expression unit for NanoLuc-mRNA containing L7Ae-specific poly(A)-surrogate (P_{hCMV}-NanoLuc-(C/D-box)$_{24}$-($BS_{shRNA-216}$)$_2$-pA::$P_{hEF1\alpha}$-Fluc-pA, pSL274), an shRNA-216 expression vector (P_{hU6}-shRNA-216; pSL4, 100ng) and different combinations of constitutive expression vectors for L7Ae-(pE59)$_2$ (pSL169) and (ERK2)$_2$-NSP3 (pSL189). Luciferase levels in culture supernatants were quantified at 48 h after the addition of 100 ng/mL recombinant human EGF. For (-) conditions, pcDNA3.1(+) was transfected instead of expression vectors. Data presented are mean ± SD of relative luciferase activity (NanoLuc/FLuc), n = 3 individual experiments.
