# Supplementary material for: Engineered poly(A)-surrogates for translational regulation and therapeutic biocomputation in mammalian cells
Source: Cell Res. 2024 Jan 4;34(1):31–46. doi: 10.1038/s41422-023-00896-y (PMC10770082; doi:10.1038/s41422-023-00896-y)
Supplement: Supplementary file 5 — Supplementary information, Fig. S5 [file 41422_2023_896_MOESM5_ESM.pdf]

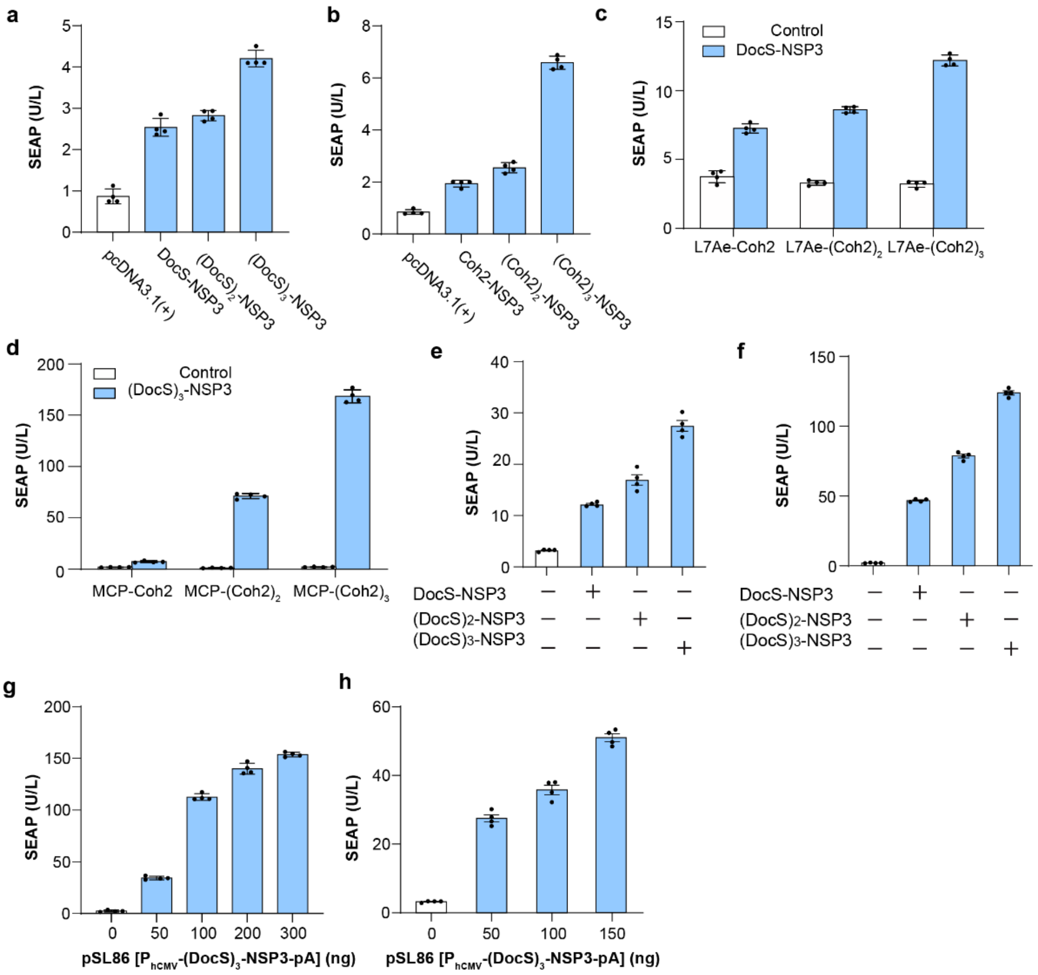

Fig. S5. Preliminary experiments for development of STIF-based protein sensors. (a, b) Optimization of NSP3-fusion constructs. (a) HEK-293 cells were co-transfected with plasmids encoding SEAP-mRNA containing an MCP-specific poly(A)-surrogate (pSL468), MCP-Coh2 (pSL674) and NSP3-fusion proteins consisting of one (pSL66), two (pSL85) or three N-terminal DocS-repeats (pSL86). SEAP expression in culture supernatants were scored at 48 h post transfection. Data are mean ± SD, n = 4. (b) HEK-293 cells were co-transfected with plasmids encoding SEAP-mRNA containing MCP-specific poly(A)-surrogate (pSL468), MCP-DocS (pSL1311), and NSP3-fusion proteins consisting of one (pSL241), two (pSL242) or three N-terminal Coh2-repeats (pSL243). SEAP expression in culture supernatants was scored at 48h post-transfection. Data are mean ±SD, n=4. **(c) Translational regulation by different C/D-box-specific DocS-tethers.** HEK-293 cells were co-transfected with plasmids encoding SEAP mRNA containing an L7Ae-specific poly(A)-surrogate

(pSL88 & pSL4), DocS-NSP3 (pSL66 or pcDNA3.1(+) as negative control) and different L7Ae-fusion proteins with one (pSL65), two (pSL82) or three C-terminal Coh2-repeats (pSL83). SEAP expression in culture supernatants were scored at 48 h post transfection. Data are mean ± SD, n = 4. **(d) Translational regulation by different MS2-box-specific DocS-tethers.** HEK-293 cells were co-transfected with plasmids encoding SEAP-mRNA containing an MCP-specific poly(A)-surrogate (pSL468), $(DocS)_3$-NSP3 (pSL86) and different MCP-fusion proteins consisting of one (pSL674), two (pSL1079) or three N-terminal Coh2-repeats (pSL1080). SEAP expression in culture supernatants were scored at 48 h post transfection. Data are mean ± SD, n = 4. **(e) Translational regulation of C/D-box-containing mRNA by different Coh2-specific eIFBPs.** HEK-293 cells were co-transfected with plasmids encoding SEAP-mRNA containing an L7Ae-specific poly(A)-surrogate (pSL88 & pSL4), L7Ae-$(Coh2)_3$ (pSL83) and NSP3-fusion proteins consisting of one (pSL66), two (pSL85) or three N-terminal DocS-repeats (pSL86). SEAP expression in culture supernatants were scored at 48 h post transfection. Data are mean ± SD, n = 4. **(f) Translational regulation of MS2-box-containing mRNA by different Coh2-specific eIFBPs.** HEK-293 cells were co-transfected with plasmids encoding SEAP-mRNA containing MCP-specific poly(A)-surrogate (pSL468), MCP-$(Coh2)_3$ (pSL1080), and NSP3-fusion proteins consisting of one (pSL66), two (pSL85) or three N-terminal DocS-repeats (pSL86). SEAP expression in culture supernatants was scored at 48h post-transfection. Data are mean ±SD, n=4. **(g, h) DocS-dependent NSP3-mediated activation of STIF-specific mRNA.** (g) For MCP-based systems, HEK-293 cells were co-transfected with plasmids encoding SEAP-mRNA containing an MCP-specific poly(A)-surrogate (pSL468), MCP-$(Coh2)_3$ (pSL1080) and different amounts of pSL86. (h) For L7Ae-based systems, HEK-293 cells were co-transfected with plasmids encoding SEAP-mRNA containing an L7Ae-specific poly(A)-surrogate (pSL88 & pSL4), L7Ae-$(Coh2)_3$ (pSL83) and different amounts of $(DocS)_3$-NSP3 expression vectors (pSL86). SEAP expression in culture supernatants were scored at 48 h post transfection. Data are mean ± SD, n = 4.
