# Supplementary material for: Engineered poly(A)-surrogates for translational regulation and therapeutic biocomputation in mammalian cells
Source: Cell Res. 2024 Jan 4;34(1):31–46. doi: 10.1038/s41422-023-00896-y (PMC10770082; doi:10.1038/s41422-023-00896-y)
Supplement: Supplementary file 6 — Supplementary information, Fig. S6 [file 41422_2023_896_MOESM6_ESM.pdf]

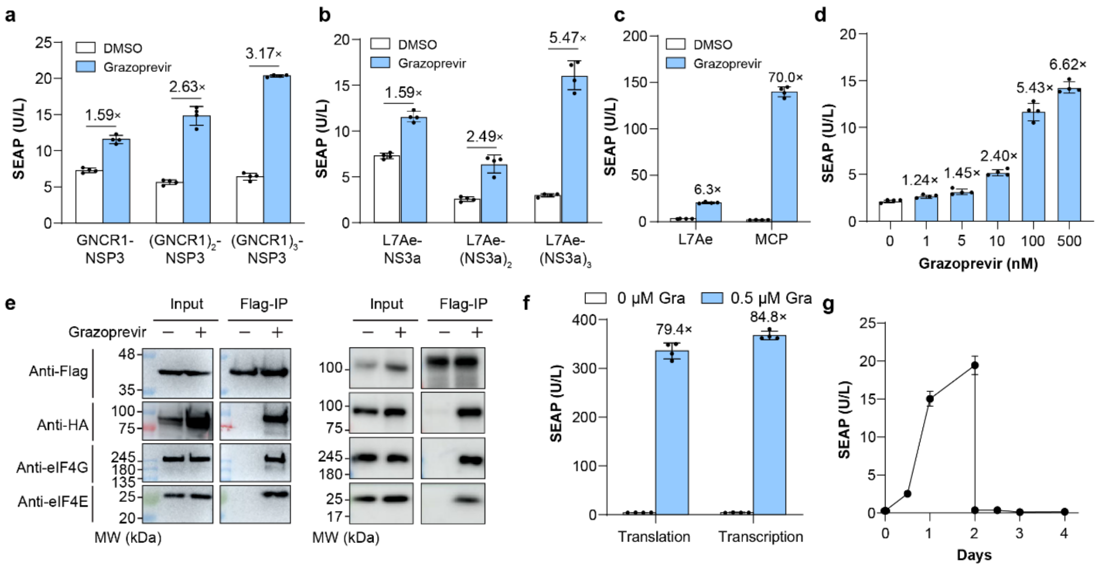

Fig. S6. Control experiments related to Figure 3. (a) Translational regulation by different grazoprevir-dependent NSP3-fusion proteins. HEK-293 cells were co-transfected with plasmids encoding SEAP-mRNA containing L7Ae-specific poly(A)-surrogate (pSL88 & pSL4), L7Ae-NS3a (pYF5) and different NSP3-fusion proteins containing one (pYF3), two (pLZ73) or three N-terminal GNCR repeats (pLZ74). SEAP levels in culture supernatants were scored at 48 h after addition of 0.1 μM grazoprevir dissolved in DMSO (vehicle control). Data are shown as the mean ± SD of n = 4 independent experiments. **(b) Translational regulation by different grazoprevir-dependent L7Ae-fusion proteins.** HEK-293 cells were co-transfected with plasmids encoding SEAP-mRNA containing L7Ae-specific poly(A)-surrogate (pSL88&pSL4), GNCR-NSP3 (pYF3) and different L7Ae-fusion proteins consisting of one (pYF5), two (pLZ75) or three C-terminal NS3a repeats (pLZ76). SEAP levels in culture supernatants were scored at 48 h after addition of 0.1 μM grazoprevir dissolved in DMSO (vehicle control). Data are shown as the mean ± SD of n = 4 independent experiments. **(c) Optimized grazoprevir-inducible regulation of SEAP translation.** For L7Ae-based systems, HEK-293 cells were co-transfected with plasmids encoding SEAP-mRNA containing C/D-box-based poly(A)-surrogate (pSL88 & pSL4), $(GNCR)_3$-NSP3 (pLZ74) and L7Ae-$(NS3a)_3$ (pLZ76). For MCP-based systems, HEK-293 cells were co-transfected with plasmids encoding SEAP mRNA

containing an MS2-box-based poly(A)-surrogate (pSL468), $(GNCR)_3$-NSP3 (pSL1032) and MCP-$(NS3a)_3$ (pSL1042). SEAP levels in culture supernatants were scored at 48 h after addition of 0.1 μM grazoprevir dissolved in DMSO (vehicle control). Data are shown as the mean ± SD of n = 4 independent experiments. **(d) Dose-dependent grazoprevir-inducible SEAP expression.** HEK-293 cells were co-transfected with plasmids encoding SEAP-mRNA containing L7Ae-specific poly(A)-surrogate (pSL88 & pSL4) and constitutive expression vectors for $(GNCR)_3$-NSP3 (pLZ74) and L7Ae-$(NS3a)_3$ (pLZ76). SEAP levels in culture supernatants were scored at 24 h after addition of different concentrations of grazoprevir. Data are shown as the mean ± SD of n = 4 independent experiments. **(e) Grazoprevir-inducible STIF-association with the endogenous eIF4F complex.** HEK-293 cells were co-transfected with expression vectors for 3xHA-tagged $(GNCR)_3$-NSP3 (pSL476) and FLAG-tagged MCP-NS3a (left, pSL1093) or L7Ae-$(NS3a)_3$ (right, pSL475) and treated with (+) or without (-) grazoprevir before immunoprecipitation. Target proteins in each lysate fraction before (input) and after immunoprecipitation (Flag-IP) were detected with anti-FLAG, anti-HA, anti-eIF4G and anti-eIF4E antibodies. Numbers on the right axis of Western blots represent molecular weights (MW) of target proteins. **(f) Endpoint performance of grazoprevir-induced translational and transcriptional gene switch switches.** For grazoprevir-inducible translation, HEK-293 cells were co-transfected with a vector encoding SEAP-mRNA containing MCP-specific poly(A)-surrogate (P_{hCMV}-SEAP-$(MS2\text{-box})_{24}$-HHR-pA, pSL468) and constitutive expression vectors for MCP-$(NS3a)_3$ (pSL503) and $(GNCR)_3$-NSP3 (pLZ74) before cultivation in cell culture medium containing 0 or 0.5 μM grazoprevir. For grazoprevir-inducible transcription, HEK-293 cells were co-transfected with a TetR-specific SEAP expression vector ($tetO_7$-$P_{hCMVmin}$-SEAP-$(C/D\text{-box})_{24}$-$(BS_{shRNA}\text{-}216)_2$-pA, pLZ79) and constitutive expression vectors for TetR-NS3a (pLZ88) and $(GNCR)_3$-VP64 (pLZ85). SEAP levels in culture supernatants were scored at 48 h after grazoprevir addition. Data show the mean ± SD of fold-changes calculated by dividing SEAP levels of grazoprevir-treated samples by SEAP levels of samples not treated with grazoprevir (n = 4 individual experiments). **(g) Durability of grazoprevir-inducible SEAP translation following mRNA delivery.**

HEK-293 cells were (co-)transfected with *in vitro*-transcribed mRNA encoding MCP-(NS3a)$_3$ (from pSL1085), (GNCR)$_3$-NSP3 (from pYW361) and SEAP-(MS2-box)$_{24}$ (from pSL468). After cultivation for 48 h in cell culture medium containing 500 nM grazoprevir, supernatants were removed and fresh medium containing 500 nM grazoprevir was supplied (arrow). SEAP levels in culture supernatants were quantified at 12 h, 24 h, 48 h, 60 h, 72 h and 96 h after first exposure to grazoprevir. Data are shown as the mean ± SD, n = 4 independent experiments.
