# Supplementary material for: Engineered poly(A)-surrogates for translational regulation and therapeutic biocomputation in mammalian cells
Source: Cell Res. 2024 Jan 4;34(1):31–46. doi: 10.1038/s41422-023-00896-y (PMC10770082; doi:10.1038/s41422-023-00896-y)
Supplement: Supplementary file 7 — Supplementary information, Fig. S7 [file 41422_2023_896_MOESM7_ESM.pdf]

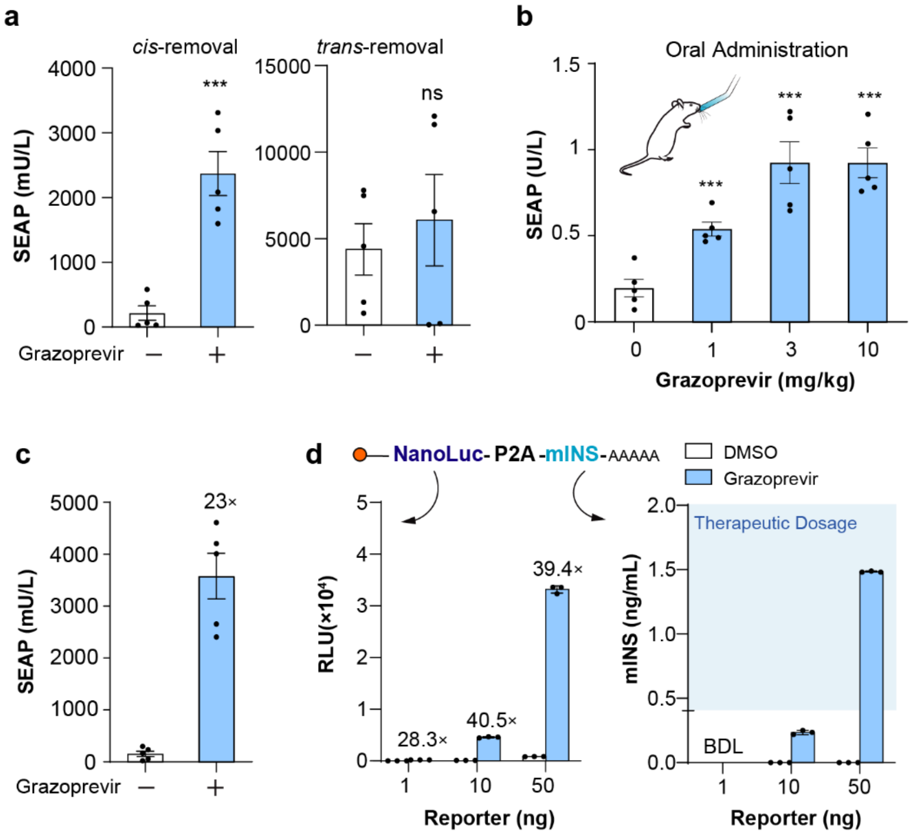

Fig. S7. ***In vivo*** **experiments related to Fig. 3. (a) Grazoprevir-inducible SEAP production in mice.** For grazoprevir-inducible SEAP production mediated by a poly(A)-surrogate created through *cis*-acting mRNA cleavage, corresponding encoding plasmid DNA consisting of pLZ76/pLZ74/pSL355 was hydrodynamically injected into the tail vein of C57BL/6 mice. For grazoprevir-inducible SEAP production mediated by a poly(A)-surrogate created through *trans*-acting mRNA cleavage, corresponding encoding plasmid DNA consisting of pLZ76/pLZ74/pSL4/pSL88 was hydrodynamically injected into the tail vein of C57BL/6 mice. After 6 h, mice received the first of 3 daily intraperitoneal injections of grazoprevir (1 mg/kg dissolved in PBS). SEAP levels in the bloodstream of mice were measured at 24 h after the first grazoprevir injection. Data are presented as the mean ± SEM of n = 5 mice per group. **(b) Dose-dependent SEAP production induced by oral grazoprevir in mice.** pLZ76 (P_{hCMV}-L7Ae-$(NS3a)_3$-pA), pLZ74 (P_{hCMV}-$(GNCR)_3$-NSP3-pA) and pSL355 (P_{hCMV}-SEAP-$(C/D\text{-}box)_{24}$-HHR-pA) were hydrodynamically injected into the tail vein of C57BL/6

mice. At 6 h post injection, mice received the first of 3 daily oral grazoprevir administrations at different doses. SEAP levels in the bloodstream of mice were measured at 24 h after the first grazoprevir administration. Data are presented as the mean ± SEM; n = 5 mice per group. **(c) Optimized grazoprevir-inducible SEAP production in mice.** Plasmid DNA consisting of pSL468/pLZ74/pSL548 was hydrodynamically injected into the tail vein of C57BL/6 mice. After 6 h, mice received the first of 3 daily intraperitoneal injections of grazoprevir (3 mg/kg dissolved in PBS). SEAP levels in the bloodstream of mice were measured at 24 h after the first grazoprevir injection. Data are presented as the mean ± SEM of n = 5 mice per group. **(d) Grazoprevir-inducible insulin expression *in vitro*.** HEK-293 cells were co-transfected with 200 ng of pSL1042 (P_{hCMV}-MCP-(NS3a)$_3$-pA), 200 ng of pSL1032 (P_{hCMV}-(GNCR)$_3$-NSP3-pA) and different amounts of pSL1003 (P_{hCMV}-NanoLuc-P2A-mINS-(MS2-box)$_{16}$-HHR-pA). NanoLuc and mINS levels in culture supernatants were scored at 48 h after addition of 0.5 µM grazoprevir. Data are mean ± SD, n = 3 independent experiments. The therapeutic efficacy window represented by physiological blood insulin levels >0.4 µg/L is marked with a blue shaded box. ns, not significant; *, p<0.1; ***, p<0.001.
