# Supplementary material for: Engineered poly(A)-surrogates for translational regulation and therapeutic biocomputation in mammalian cells
Source: Cell Res. 2024 Jan 4;34(1):31–46. doi: 10.1038/s41422-023-00896-y (PMC10770082; doi:10.1038/s41422-023-00896-y)
Supplement: Supplementary file 8 — Supplementary information, Fig. S8 [file 41422_2023_896_MOESM8_ESM.pdf]

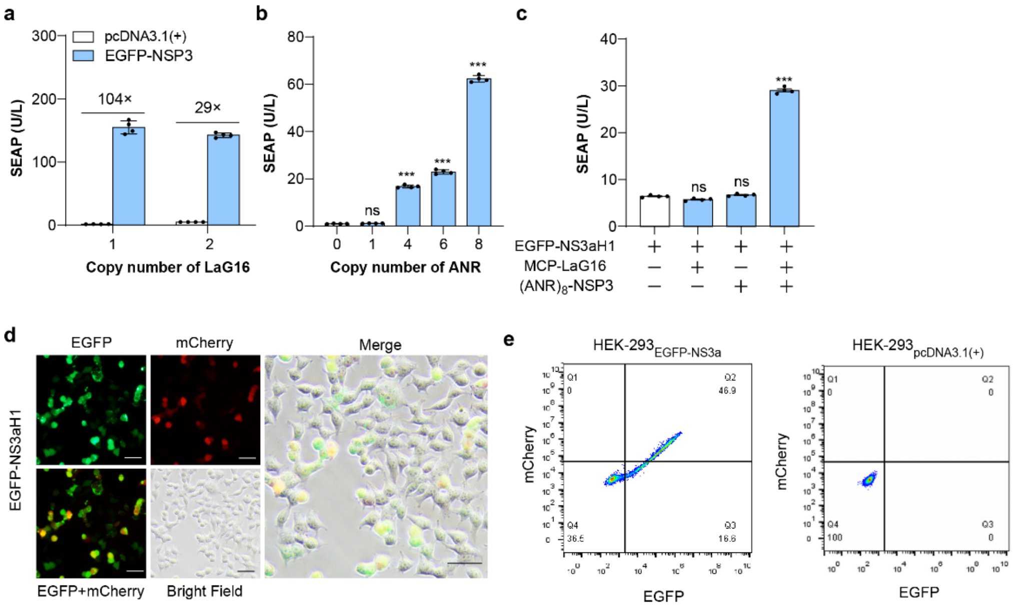

Fig. S8. Control experiments related to Figure 4. (a) Translational regulation by different EGFP-specific MCP-fusion proteins. HEK-293 cells were co-transfected with plasmids encoding SEAP mRNA containing an MCP-specific poly(A)-surrogate (pSL468), a constitutive EGFP-NSP3 expression vector (pSL942) and expression vectors for different MCP-LaG16 variants containing one (pSL776) or two (pSL777) tandem LaG16 repeats. Transfection of pcDNA3.1(+) instead of pSL942 was used as a negative control. SEAP expression in the culture supernatant were profiled at 48 h after transfection. Data presented are mean ± SD, n = 4. **(b) Translational regulation by different NS3a(H1)-specific NSP3-fusion proteins.** HEK-293 cells were co-transfected with plasmids encoding SEAP-mRNA containing MCP-specific poly(A)-surrogate (pSL468), a constitutive MCP-(NS3a(H1))$_3$ expression vector (pSL548) and expression vectors for different ANR-NSP3 variants containing different tandem ANR repeats (ANR-NSP3, pSL704; (ANR)$_2$-NSP3, pSL549; (ANR)$_6$-NSP3, pSL581; (ANR)$_8$-NSP3, pSL582). SEAP expression in the culture supernatant were profiled at 48 h after transfection. Data presented are mean ± SD, n = 4. **(c) Construction of an EGFP-NS3a(H1) sensor in mammalian cells.** HEK-293 cells were co-transfected with plasmids encoding reporter SEAP-mRNA containing MCP-specific poly(A)-surrogate (pSL468), a constitutive EGFP-NS3a(H1) expression vector (pSL775) and different combinations of MCP-LaG16 (pSL776) and (ANR)$_8$-NSP3expression vectors (pSL582). Transfection of pcDNA3.1(+) instead of pSL776 and/or pSL582 was used

as a negative control (-). SEAP expression in the culture supernatant were profiled at 48 h after transfection. Data presented are mean ± SD, n = 4. **(d, e) Target-specificity of genetically encoded EGFP-NS3a(H1) protein sensor.** HEK-293 cells were co-transfected with plasmids encoding mCherry-mRNA containing MCP-specific poly(A)-surrogate (pSL683, P_{hCMV}-NanoLuc-P2A-mCherry-(MS2-box)$_{24}$-HHR-pA) and constitutive expression vectors for MCP-LaG16 (pSL776), (ANR)$_8$-NSP3 (pSL582) and EGFP-NS3a(H1) (pSL775). Transfection of pcDNA3.1(+) instead of pSL775 was used as a negative control. At 48 h post transfection, **(d)** fluorescent images showing co-localized EGFP/mCherry signals were acquired (scale bar: 50 µm) before **(e)** flow-cytometric analysis was performed (10'000 cells per group). Representative data from 3 independent experiments are shown. Bars represent the mean and SD, and filled circles show individual results. Numbers above bars are fold change. ns, not significant; ***, $p<0.001$.
