# Supplementary material for: Engineered poly(A)-surrogates for translational regulation and therapeutic biocomputation in mammalian cells
Source: Cell Res. 2024 Jan 4;34(1):31–46. doi: 10.1038/s41422-023-00896-y (PMC10770082; doi:10.1038/s41422-023-00896-y)
Supplement: Supplementary file 9 — Supplementary information, Fig. S9 [file 41422_2023_896_MOESM9_ESM.pdf]

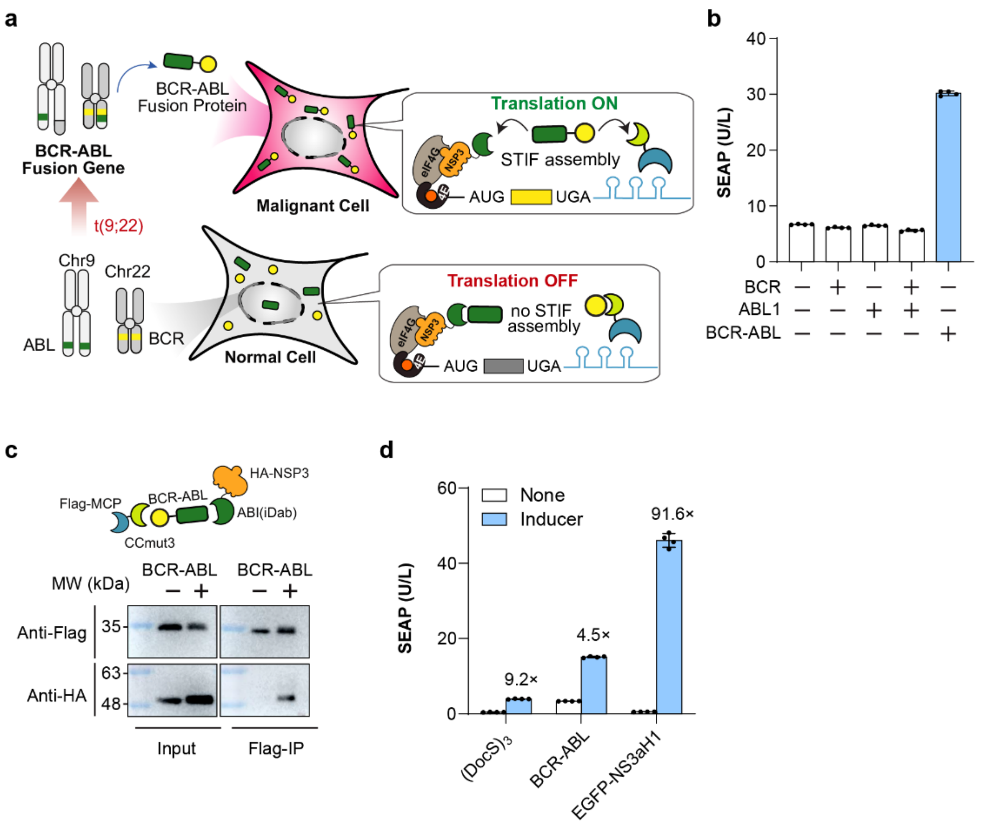

Fig. S9. Engineering of a STIF-based sensor for cancer-related fusion genes. (a) Engineering of a STIF-based sensor for the BCR-ABL fusion protein. In chronic myelogenous leukemia (CML), chromosomal translocation genetically fuses the BCR gene at 22q11 with the ABL1 tyrosine kinase-encoding gene at 9q34, forming a hybrid oncoprotein BCR–ABL with increased kinase activity. Bipartite STIFs where each split-component is engineered to contain an intrabody that specifically binds each individual BCR or ABL1 protein in its native form can only be reconstituted to activate STIF-dependent translation in cells containing the fusion protein configuration. **(b) Selectivity of the BCR-ABL fusion protein sensor.** HEK-293 cells were co-transfected with a SEAP-producing BCR-ABL sensor (constitutive expression of MCP-ABI(iDab) (pSL860), CCmut3-NSP3 (pSL863) and MCP-specific SEAP-mRNA) and expression vectors for either BCR (pSL1045), ABL (pSL1046) or BCR-ABL (pSL1014). SEAP expression in the culture supernatant were profiled at 48h after transfection. Data presented are mean± SD, n = 4 individual experiments. **(c) BCR-ABL-mediated association of MCP-ABI(iDab) and CCmut3-NSP3.** HEK-293 cells were co-transfected with expression vectors for 3xFLAG-tagged MCP-ABI(iDab)

(pSL1101), 3xHA-tagged CCmut3-NSP3 and BCR-ABL (+, pSL1014) or pcDNA3.1(+) (-, negative control) at 48h before immunoprecipitation. Target proteins in each lysate fraction before (input) and after immunoprecipitation (Flag-IP) were detected with anti-FLAG and anti-HA antibodies. Numbers on the right axis of Western blots represent molecular weights (MW) of target proteins. **(d) Comparative analysis of STIF-based protein sensors.** For detection of overexpressed BCR-ABL (transfection of 0 or 50 ng of pSL1014), HEK-293 cells were co-transfected with constitutive expression vectors for MCP-ABI(iDab)$_3$ (pSL860) and CCmut3-NSP3 (pSL863). For detection of overexpressed (DocS)$_3$ (transfection of 0 or 50 ng of pSL244), HEK-293 cells were co-transfected with constitutive expression vectors for MCP-(Coh2)$_3$ (pSL1080) and (Coh2)$_3$-NSP3 (pSL243). For detection of overexpressed EGFP-NS3aH1 (transfection of 0 or 50 ng of pSL775), HEK-293 cells were co-transfected with constitutive expression vectors for MCP-(LaG16)$_2$ (pSL776) and (ANR)$_8$-NSP3 (pSL582). SEAP-mRNA with MCP-specific poly(A)-surrogates (P_{hCMV}-SEAP-(MS2)$_{24}$-HHR-pA, pSL468) was used as the reporter vector in all cases. SEAP expression in culture supernatants was scored at 48 h post-transfection. Data is the mean ± SD of n = 4 independent experiments.
