# Supplementary material for: Engineered poly(A)-surrogates for translational regulation and therapeutic biocomputation in mammalian cells
Source: Cell Res. 2024 Jan 4;34(1):31–46. doi: 10.1038/s41422-023-00896-y (PMC10770082; doi:10.1038/s41422-023-00896-y)
Supplement: Supplementary file 10 — Supplementary information, Fig. S10 [file 41422_2023_896_MOESM10_ESM.pdf]

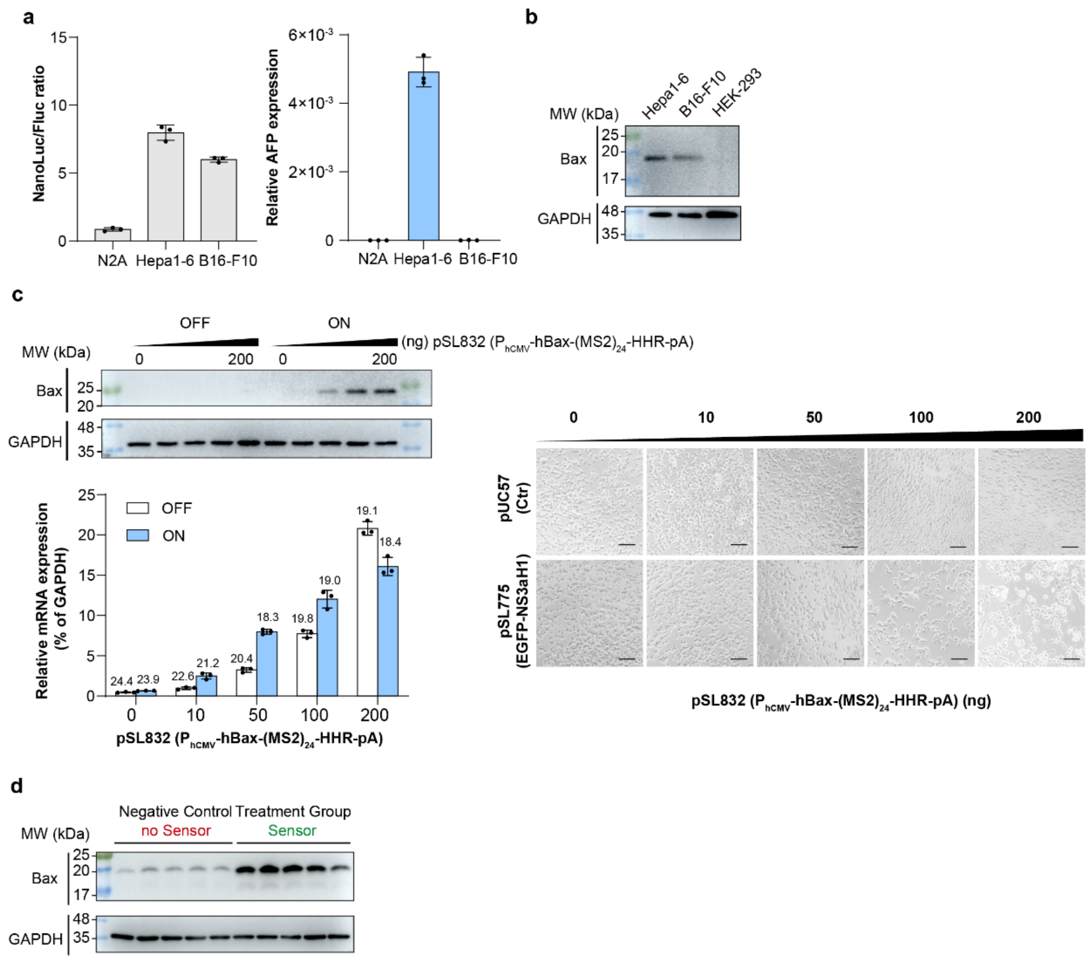

Fig. S10. Specificity and safety of tissue-specific promoters (TSP) and protein sensors. (a) Quantification of AFP-levels using (left panel) TSP-driven reporter gene expression and (right panel) qRT-PCR. (left panel) N2A, Hepa1-6 and B16-F10 cells were co-transfected with a P_{MusAFP}-driven NanoLuc expression vector (pSLM54) and a constitutive FLuc expression vector (pYW99). Luciferase levels were quantified at 48 h after transfection. Data presented are mean ± SD of relative luciferase activity (NanoLuc/FLuc) for n = 3 individual experiments. (right panel) Relative AFP expression levels in N2A, Hepa1-6 and B16-F10 cells were profiled by qRT-PCR using murine AFP-specific primers listed in **Supplementary information, Table S1** and normalized to the gene encoding murine ribosomal protein (Rplp0). Data presented are mean ± SD, n = 3. Bars represent the mean and SD, and filled circles show individual results. **(b) Western Blot analysis of endogenous Bax and GAPDH levels in various mammalian cell lines. (c) Therapeutic efficacy window of hBax overexpression *in***

vitro. HEK-293 cells were transfected with constitutive expression vectors for MCP-LaG16 (pSL776), $(ANR)_8$-NSP3 (pSL582) and different amounts of an expression vector for hBax-mRNA with MCP-specific poly(A)-surrogate (P_{hCMV}-hBax-$(MS2)_{24}$-HHR-pA, pSL832). For ON-state conditions, cells were further transfected with a constitutive EGFP-NS3a(H1) expression vector (pSL775) driving assembly of MCP-LaG16 and $(ANR)_8$-NSP3 to form a closed-loop mRNA configuration. For OFF-state conditions, cells were transfected with pUC57 instead of pSL775. Relative protein and mRNA levels of total Bax expression as well as microscopic images of transfected cells (scale bar: 100 µm) were assessed at 24 hours post-transfection. Data of qRT-PCR show the mean of absolute C_T-values of Bax mRNA as well as relative transcript levels normalized to endogenous GAPDH expression (C_T (GAPDH) = 15.9 ± 0.7 for ON-state samples; C_T (GAPDH) = 16.1 ± 0.5 for OFF-state samples), n = 3 independent experiments. Western Blot analysis was performed using an anti-Bax antibody. **(d) Immunoblots of tumor lysates as described in Fig. 6e.**
