# Supplementary material for: Engineered poly(A)-surrogates for translational regulation and therapeutic biocomputation in mammalian cells
Source: Cell Res. 2024 Jan 4;34(1):31–46. doi: 10.1038/s41422-023-00896-y (PMC10770082; doi:10.1038/s41422-023-00896-y)
Supplement: Supplementary file 11 — Supplementary information, Table S1 [file 41422_2023_896_MOESM11_ESM.pdf]

Table S1. qRT-PCR primers used in this study

EGFP-fw	CCACATGAAGCAGCACGACTT
EGFP-rev	GGTGCGCTCCTGGACGTA
GAPDH-fw	ACATCGCTCAGACACCATG
GAPDH-rev	TGTAGTTGAGGTCAATGAA
murine AFP-fw	ATTCCTCCCAGTGCGTGACGGA
murine AFP-rev	TGCGTGCCAGCAGACACTGATG
Rplp0-fw	GAAACTGCTGCCTCACATCCG
Rplp0-rev	GCTGGCACAGTGACCTCACACG
SEAP-fw	GGCTCTGTCCAAGACATACAA
SEAP-rev	GTCGTGTTGCACTGGTTAAAG
HusBax-fw	AGCTCTGAGCAGATCATGAAGA
HusBax-rev	AGTTGAAGTTGCCGTCAGAAAA
