# Supplementary material for: Engineered poly(A)-surrogates for translational regulation and therapeutic biocomputation in mammalian cells
Source: Cell Res. 2024 Jan 4;34(1):31–46. doi: 10.1038/s41422-023-00896-y (PMC10770082; doi:10.1038/s41422-023-00896-y)
Supplement: Supplementary file 12 — Supplementary information, Table S2 [file 41422_2023_896_MOESM12_ESM.pdf]

Table S2. Plasmids designed and used in this study

Plasmid	Description and Cloning Strategy	Reference or source
pcDNA3.1(+)	Mammalian expression vector (P_{hCMV}-MCS-pA).	Invitrogen, CA
pUC57	Promoterless cloning vector for ampicillin selection.	GenScript, NJ
AAV-CAG-EGFP	AAV vector for P_{CAG}-driven constitutive EGFP expression (P_{CAG}-EGFP-pA)	Addgene no. 28014 (ref.[1])
pAdDeltaF6	AAV helper plasmid expressing E4, E2a and VA.	Addgene no. 112867
pAAV2/8	AAV packaging plasmid expressing Rep2/Cap8 genes.	Addgene no. 112864
pCMV-T7-SB100	Mammalian P_{hCMV}-driven constitutive SB100X expression vector (P_{hCMV}-SB100X-pA).	Addgene no. 34879
pMF111	Mammalian TetR-specific SEAP expression vector ($P_{hCMV*-1}$-SEAP-pA).	(ref.[2])
pSA776	Mammalian P_{SV40}-driven constitutive λN-mCherry expression vector (P_{SV40}-λN-mCherry-pA).	(ref.[3])
pWS164	Mammalian P_{hCMV}-driven constitutive EGFP expression vector (P_{hCMV}-EGFP-pA).	(ref.[4]**)**
pWH127	Mammalian P_{hCMV}-driven constitutive eIF4G-2CaM-M13-L7Ae expression vector (P_{hCMV}-eIF4G-2CaM-M13-L7Ae-pA).	This work
pMX116	Mammalian P_{SV40}-driven SEAP expression vector containing λN-repressible HHR in the 3'-UTR (P_{SV40}-SEAP-HHR-pA).	This work
pMX331	Mammalian $P_{hEF1\alpha}$-driven constitutive FKBP-L7Ae expression vector ($P_{hEF1\alpha}$-FKBP-L7Ae-pA).	This work
pYW99	Mammalian P_{hCMV}-driven constitutive FLuc expression vector (P_{hCMV}-FLuc-pA).	This work
pYW361	P_{T7}-driven expression vector for *in vitro* transcription of $(GNCR)_3$-NSP3 mRNA containing stabilized 5'-UTR, 3'-UTR and an extended poly(A) signal (P_{T7}-5'UTR*-$(GNCR)_3$-NSP3-3'UTR*-A_{92}).	This work

pYF3	Mammalian P_{hCMV}-driven constitutive GNCR-NSP3 expression vector (P_{hCMV}-GNCR-NSP3-pA).	This work
pYF5	Mammalian P_{hCMV}-driven constitutive L7Ae-NS3a expression vector (P_{hCMV}-L7Ae-NS3a-pA).	This work
pLZ16	Mammalian P_{hCMV}-driven constitutive PABP-L7Ae expression vector (P_{hCMV}-PABP-L7Ae-pA).	This work
pLZ27	Mammalian $P_{hEF1\alpha}$-driven constitutive L7Ae-NSP3 expression vector ($P_{hEF1\alpha}$-L7Ae-NSP3-pA).	This work
pLZ42	Mammalian $P_{hEF1\alpha}$-driven constitutive FRB-NSP3 expression vector ($P_{hEF1\alpha}$-FRB-NSP3-pA).	This work
pLZ55	Mammalian P_{hCMV}-driven constitutive L7Ae-CIB1 expression vector (P_{hCMV}-L7Ae-CIB1-pA).	This work
pLZ68	Mammalian P_{hCMV}-driven constitutive Cry2-NSP3 expression vector (P_{hCMV}-Cry2-NSP3-pA).	This work
pSL71	Mammalian P_{hCMV}-driven constitutive Cry2-NSP3 expression vector (P_{hCMV}-Cry2-NSP3-pA).	This work
pLZ72	Mammalian P_{hCMV}-driven constitutive $(DNCR2)_3$-NSP3 expression vector (P_{hCMV}-$(DNCR2)_3$-NSP3-pA).	This work
pLZ73	Mammalian P_{hCMV}-driven constitutive $(GNCR)_2$-NSP3 expression vector (P_{hCMV}-$(GNCR)_2$-NSP3-pA).	This work
pLZ74	Mammalian P_{hCMV}-driven constitutive $(GNCR)_3$-NSP3 expression vector (P_{hCMV}-$(GNCR)_3$-NSP3-pA).	This work
pLZ75	Mammalian P_{hCMV}-driven constitutive L7Ae-$(NS3a)_2$ expression vector (P_{hCMV}-L7Ae-$(NS3a)_2$-pA).	This work

pLZ76	Mammalian P_{hCMV}-driven constitutive L7Ae-(NS3a)$_3$ expression vector (P_{hCMV}-L7Ae-(NS3a)$_3$-pA).	This work
pLZ79	Mammalian TetR-specific expression vector for shRNA-216-repressible SEAP-mRNA containing an L7Ae-specific poly(A)-surrogate ($P_{hCMV*-1}$-SEAP-(C/Dbox)$_{24}$-BS (shRNA-216)$_2$-pA).	This work
pLZ85	Mammalian P_{hCMV}-driven constitutive (GNCR)$_3$-VP64 expression vector (P_{hCMV}-(GNCR)$_3$-VP64-pA).	This work
pLZ88	Mammalian P_{hCMV}-driven constitutive TetR-NS3a expression vector (P_{hCMV}-TetR-NS3a-pA).	This work
pLZ248	Mammalian P_{hCMV}-driven constitutive L7Ae-VPg expression vector (P_{hCMV}-L7Ae-VPg-pA).	This work
pLZ311	Mammalian P_{hCMV}-driven constitutive DocS-VPg expression vector (P_{hCMV}-DocS-VPg-pA).	This work
pLZ312	Mammalian P_{hCMV}-driven constitutive DocS-eIF4E^{K119A} expression vector (P_{hCMV}-DocS-eIF4E^{K119A}-pA).	This work
pLZ323	Mammalian P_{SV40}-driven SEAP expression vector containing a MALAT1 and HHR motif in the 3'-UTR (P_{SV40}-SEAP-MALAT1-HHR-pA).	This work
pLZ324	Mammalian P_{hCMV}-driven SEAP expression vector containing a MALAT1 motif in the 3'-UTR (P_{hCMV}-SEAP-MALAT1-pA).	This work
pSL4	Mammalian P_{hU6}-driven shRNA-216 expression vector (P_{hU6}-shRNA-216).	This work
pSL31	Mammalian P_{hCMV}-driven expression vector for shRNA-216-repressible L7Ae-specific SEAP-mRNA (P_{hCMV}-SEAP-(C/Dbox)$_8$-BS(shRNA-216)$_2$-pA).	This work
pSL44	Mammalian P_{hCMV}-driven constitutive Coh2-L7Ae expression vector (P_{hCMV}-Coh2-L7Ae-pA).	This work

pSL47	Mammalian P_{hCMV}-driven constitutive PABP-DocS expression vector (P_{hCMV}-PABP-DocS-pA).	This work
pSL65	Mammalian P_{hCMV}-driven constitutive L7Ae-Coh2 expression vector (P_{hCMV}-L7Ae-Coh2-pA).	This work
pSL66	Mammalian P_{hCMV}-driven constitutive DocS-NSP3 expression vector (P_{hCMV}-DocS-NSP3-pA).	This work
pSL71	Mammalian P_{hCMV}-driven constitutive Cry2-NSP3 expression vector (P_{hCMV}-Cry2-NSP3-pA).	This work
pSL80	Mammalian P_{hCMV}-driven expression vector for shRNA-216-repressible L7Ae-specific SEAP-mRNA (P_{hCMV}-SEAP-(C/Dbox)$_{16}$-BS (shRNA-216)$_2$-pA).	This work
pSL81	Mammalian P_{hCMV}-driven expression vector for shRNA-216-repressible L7Ae-specific SEAP-mRNA (P_{hCMV}-SEAP-(C/Dbox)$_{12}$-BS(shRNA-216)$_2$-pA).	This work
pSL82	Mammalian P_{hCMV}-driven constitutive L7Ae-(Coh2)$_2$ expression vector (P_{hCMV}-L7Ae-(Coh2)$_2$-pA).	This work
pSL83	Mammalian P_{hCMV}-driven constitutive L7Ae-(Coh2)$_3$ expression vector (P_{hCMV}-L7Ae-(Coh2)$_3$-pA).	This work
pSL85	Mammalian P_{hCMV}-driven constitutive (DocS)$_2$-NSP3 expression vector (P_{hCMV}-(DocS)$_2$-NSP3-pA).	This work
pSL86	Mammalian P_{hCMV}-driven constitutive (DocS)$_3$-NSP3 expression vector (P_{hCMV}-(DocS)$_3$-NSP3-pA).	This work
pSL87	Mammalian P_{hCMV}-driven constitutive DocS-eIF4G expression vector (P_{hCMV}-DocS-eIF4G-pA).	This work
pSL88	Mammalian P_{hCMV}-driven expression vector for shRNA-216-repressible L7Ae-specific SEAP-mRNA (P_{hCMV}-SEAP-(C/Dbox)$_{24}$-BS(shRNA-216)$_2$-pA).	This work

pSL95	Mammalian P_{hCMV}-driven constitutive MCP-NSP3 expression vector (P_{hCMV}-MCP-NSP3-pA).	This work
pSL154	Mammalian P_{hCMV}-driven constitutive eIF4G-MCP expression vector (P_{hCMV}-eIF4G-MCP-pA).	This work
pSL169	Mammalian P_{hCMV}-driven constitutive L7Ae-$(pE59)_2$ expression vector (P_{hCMV}-L7Ae-$(pE59)_2$-pA).	This work
pSL189	Mammalian P_{hCMV}-driven constitutive $(ERK2)_2$-NSP3 expression vector (P_{hCMV}-$(ERK2)_2$-NSP3-pA).	This work
pSL243	Mammalian P_{hCMV}-driven constitutive $(Coh2)_3$-NSP3 expression vector (P_{hCMV}-$(Coh2)_3$-NSP3-pA).	This work
pSL244	Mammalian P_{hCMV}-driven constitutive $(DocS)_3$ expression vector (P_{hCMV}-$(DocS)_3$-pA).	This work
pSL260	Mammalian P_{hU6}-driven expression vector for EGFP-specific shRNA (P_{hU6}-shR-EGFP).	This work
pSL274	Mammalian dual reporter vector containing a constitutive FLuc expression unit and a constitutive expression unit for shRNA-216-repressible NanoLuc-mRNA with an L7Ae-specific poly(A)-surrogate (P_{hCMV}-NanoLuc-$(C/Dbox)_{24}$-BS$(shRNA\text{-}216)_2$-pA::$P_{hEF1\alpha}$-FLuc-pA).	This work
pSL334	Mammalian P_{hCMV}-driven constitutive λN-Coh2 expression vector (P_{hCMV}-λN-Coh2-pA).	This work
pSL355	Mammalian P_{hCMV}-driven expression vector for SEAP-mRNA containing an L7Ae-specific poly(A)-surrogate (P_{hCMV}-SEAP-$(C/Dbox)_{24}$-HHR-pA).	This work
pSL435	Mammalian P_{hCMV}-driven constitutive MCP-EGFP expression vector (P_{hCMV}-MCP-EGFP-pA).	This work

pSL446	AAV vector for P_{hCMV}-driven constitutive $(GNCR)_3$-NSP3 expression (P_{hCMV}-$(GNCR)_3$-NSP3-pA).	This work
pSL468	Mammalian P_{hCMV}-driven expression vector for SEAP-mRNA containing an MCP-specific poly(A)-surrogate (P_{hCMV}-SEAP-$(MS2\text{-}box)_{24}$-HHR-pA).	This work
pSL475	Mammalian P_{hCMV}-driven constitutive L7Ae-$(NS3a)_3$-3xFLAG expression vector (P_{hCMV}-L7Ae-$(NS3a)_3$-3xFLAG-pA).	This work
pSL476	Mammalian P_{hCMV}-driven constitutive 3xHA-$(GNCR)_3$-NSP3 expression vector (P_{hCMV}-3xHA-$(GNCR)_3$-NSP3-pA).	This work
pSL503	Mammalian P_{hCMV}-driven constitutive MCP-$(NS3a)_3$ expression vector (P_{hCMV}-MCP-$(NS3a)_3$-pA).	This work
pSL511	Mammalian P_{hCMV}-driven constitutive expression vector for SEAP-mRNA containing an MCP-specific poly(A)-surrogate (P_{hCMV}-SEAP-$(MS2\text{-}box)_{24}$-HHR-pA).	This work
pSL512	AAV vector for P_{hCMV}-driven constitutive MCP-$(NS3a)_3$ expression (P_{hCMV}-MCP-$(NS3a)_3$-pA).	This work
pSL515	Mammalian P_{hCMV}-driven expression vector for SEAP-mRNA containing MCP-specific poly(A)-surrogate with 8 tandem MS2-box repeats (P_{hCMV}-SEAP-$(MS2\text{-}box)_8$-HHR-pA).	This work
pSL516	Mammalian P_{hCMV}-driven expression vector for SEAP-mRNA containing an MCP-specific poly(A)-surrogate (P_{hCMV}-SEAP-$(MS2\text{-}box)_{16}$-HHR-pA).	This work
pSL517	Mammalian P_{hCMV}-driven constitutive SEAP expression vector (P_{hCMV}-SEAP-pA).	This work
pSL548	Mammalian P_{hCMV}-driven constitutive MCP-$(NS3a(H1))_3$ expression vector (P_{hCMV}-MCP-$(NS3a(H1))_3$-pA).	This work
pSL549	Mammalian P_{hCMV}-driven constitutive $(ANR)_4$-NSP3 expression vector (P_{hCMV}-$(ANR)_4$-NSP3-pA).	This work

pSL581	Mammalian P_{hCMV}-driven constitutive $(ANR)_6$-NSP3 expression vector (P_{hCMV}-$(ANR)_6$-NSP3-pA).	This work
pSL582	Mammalian P_{hCMV}-driven constitutive $(ANR)_8$-NSP3 expression vector (P_{hCMV}-$(ANR)_8$-NSP3-pA).	This work
pSL615	Mammalian P_{hCMV}-driven constitutive Bcl-XL-NSP3 expression vector (P_{hCMV}-Bcl-XL-NSP3-pA).	This work
pSL637	Mammalian P_{hCMV}-driven constitutive MCP-$(pE59)_2$ expression vector (P_{hCMV}-MCP-$(pE59)_2$-pA).	This work
pSL661	Mammalian P_{hCMV}-driven constitutive L7Ae-LD3 expression vector (P_{hCMV}-L7Ae-LD3-pA).	This work
pSL667	Mammalian P_{hCMV}-driven constitutive L7Ae-LD1 expression vector (P_{hCMV}-L7Ae-LD1-pA).	This work
pSL674	Mammalian P_{hCMV}-driven constitutive MCP-Coh2 expression vector (P_{hCMV}-MCP-Coh2-pA).	This work
pSL683	Mammalian P_{hCMV}-driven constitutive expression vector for NanoLuc-P2A-mCherry-mRNA containing an MCP-specific poly(A)-surrogate (P_{hCMV}-NanoLuc-P2A-mCherry-$(MS2\text{-}box)_{24}$-HHR-pA).	This work
pSL685	Mammalian P_{hCMV}-driven constitutive expression vector for NanoLuc-P2A-mINS-mRNA containing an MCP-specific poly(A)-surrogate (P_{hCMV}-NanoLuc-P2A-mINS-$(MS2\text{-}box)_{24}$-HHR-pA).	This work
pSL688	SB100X-specific transposon containing a constitutive expression unit for NanoLuc-P2A-mINS-mRNA with an MCP-specific poly(A)-surrogate and a constitutive expression unit for PuroR (ITR-P_{RPBSA}-PuroR-pA:P_{hCMV}-NanoLuc-P2A-mINS-$(MS2\text{-}box)_{24}$-HHR-pA-ITR).	This work

pSL703	Mammalian P_{hCMV}-driven constitutive L7Ae-NS3a(H1) expression vector (P_{hCMV}-L7Ae-NS3a(H1)-pA).	This work
pSL704	Mammalian P_{hCMV}-driven constitutive ANR-NSP3 expression vector (P_{hCMV}-ANR-NSP3-pA).	This work
pSL721	SB100X-specific transposon containing constitutive expression units of ZeoR and MCP-(NS3a)$_3$ (ITR-P_{RPBSA}-ZeoR-pA:P_{hCMV}-MCP-(NS3a)$_3$-pA-ITR).	This work
pSL722	SB100X-specific transposon containing constitutive expression units of PuroR and (GNCR)$_3$-NSP3 (ITR-P_{RPBSA}-PuroR-pA:P_{hCMV}-(GNCR)$_3$-NSP3-pA-ITR)	This work
pSL762	Mammalian P_{hCMV}-driven constitutive 3xFLAG-L7Ae-NSP3 expression vector (P_{hCMV}-3xFLAG-L7Ae-NSP3-pA).	This work
pSL763	Mammalian P_{hCMV}-driven constitutive PABP-L7Ae-3xFLAG expression vector (P_{hCMV}-PABP-L7Ae-3xFLAG-pA).	This work
pSL767	Mammalian P_{hCMV}-driven constitutive expression vector for SEAP-mRNA with self-cleavable polyA (P_{hCMV}-SEAP-HHR$_2$-pA).	This work
pSL768	Mammalian P_{hCMV}-driven constitutive expression vector for SEAP-mRNA with self-cleavable polyA (P_{hCMV}-SEAP-HHR$_4$-pA).	This work
pSL775	AAV vector for $P_{hEF1\alpha}$-driven constitutive EGFP-NS3a(H1) expression ($P_{hEF1\alpha}$-EGFP-NS3a(H1)-pA).	This work
pSL776	Mammalian P_{hCMV}-driven constitutive MCP-LaG16 expression vector (P_{hCMV}-MCP-LaG16-pA).	This work
pSL777	Mammalian P_{hCMV}-driven constitutive MCP-(LaG16)$_2$ expression vector (P_{hCMV}-MCP-(LaG16)$_2$-pA).	This work
pSL781	Mammalian P_{hCMV}-driven expression vector for 3xFLAG-FLuc-mRNA containing MCP-specific poly(A)-surrogate with 24 tandem MS2-box repeats (P_{hCMV}-3xFLAG-FLuc-(MS2-box)$_{24}$-HHR-pA).	This work

pSL796	Mammalian P_{hCMV}-driven constitutive SP-EGFP-NS3a(H1) expression vector (P_{hCMV}-SP-EGFP-NS3a(H1)-pA).	This work
pSL797	Mammalian P_{hCMV}-driven constitutive NLS-EGFP-NS3a(H1) expression vector (P_{hCMV}-NLS-EGFP-NS3a(H1)-pA).	This work
pSL798	Mammalian P_{hCMV}-driven constitutive TM-EGFP-NS3a(H1) expression vector (P_{hCMV}-TM-EGFP-NS3a(H1)-pA).	This work
pSL799	Mammalian P_{hCMV}-driven constitutive EGFP-NS3a(H1)-CAAX expression vector (P_{hCMV}-EGFP-NS3a(H1)-CAAX-pA).	This work
pSL813	Mammalian P_{MusAFP}-driven expression vector for NanoLuc-P2A-mCherry-mRNA containing an MCP-specific poly(A)-surrogate (P_{MusAFP}-NanoLuc-P2A-mCherry-(MS2-box)$_{24}$-HHR-pA).	This work
pSL816	SB100X-specific transposon containing constitutive expression units for EGFP-NS3a(H1), BFP and PuroR (ITR-$P_{hEF1\alpha}$-EGFP-NS3a(H1)-pA:P_{RPBSA}-BFP-P2A-PuroR-pA-ITR).	This work
pSL818	Mammalian P_{hCMV}-driven constitutive NS3a(H1) expression vector (P_{hCMV}-NS3a(H1)-pA).	This work
pSL824	Mammalian P_{hCMV}-driven constitutive NES-EGFP-NS3a(H1) expression vector (P_{hCMV}-NES-EGFP-NS3a(H1)-pA).	This work
pSL831	Mammalian P_{hCMV}-driven expression vector for mBax-mRNA containing MCP-specific poly(A)-surrogate with 24 tandem MS2-box repeats (P_{hCMV}-mBax-(MS2-box)$_{24}$-HHR-pA).	This work
pSL832	Mammalian P_{hCMV}-driven expression vector for hBax-mRNA containing MCP-specific poly(A)-surrogate with 24 tandem MS2-box repeats (P_{hCMV}-hBax-(MS2-box)$_{24}$-HHR-pA).	This work

pSL834	Mammalian P_{hCMV}-driven constitutive TetR-LaG16 expression vector (P_{hCMV}-TetR-LaG16-pA).	This work
pSL836	Mammalian P_{hCMV}-driven constitutive $(ANR)_8$-VP64 expression vector (P_{hCMV}-$(ANR)_8$-VP64-pA).	This work
pSL857	Mammalian P_{MusAFP}-driven expression vector for SEAP-mRNA containing an MCP-specific poly(A)-surrogate (P_{MusAFP}-SEAP-$(MS2\text{-}box)_{24}$-HHR-pA).	This work
pSL860	Mammalian P_{hCMV}-driven constitutive MCP-ABI$(iDab)_3$ expression vector (P_{hCMV}-MCP-ABI$(iDab)_3$-pA).	This work
pSL863	Mammalian P_{hCMV}-driven constitutive CCmut3-NSP3 expression vector (P_{hCMV}-CCmut3-NSP3-pA).	This work
pSL875	Mammalian P_{hCMV}-driven constitutive MCP-LaM8_AK47 expression vector (P_{hCMV}-MCP-LaM8_AK47-pA).	This work
pSL876	Mammalian P_{hCMV}-driven constitutive mCherry-NSP3 expression vector (P_{hCMV}-mCherry-NSP3-pA).	This work
pSL886	Mammalian P_{MusAFP}-driven expression vector for mBax-mRNA containing an MCP-specific poly(A)-surrogate (P_{MusAFP}-mBax-$(MS2\text{-}box)_{24}$-HHR-pA).	This work
pSL901	Mammalian P_{hCMV}-driven constitutive DrBPhP-NSP3 expression vector (P_{hCMV}-DrBPhP-NSP3-pA).	This work
pSL917	Mammalian P_{hCMV}-driven constitutive MCP-$(Aff6\_V18F\Delta N)_4$ expression vector (P_{hCMV}-MCP-$(Aff6\_V18F\Delta N)_4$-pA).	This work
pSL942	Mammalian P_{hCMV}-driven constitutive EGFP-NSP3 expression vector (P_{hCMV}-EGFP-NSP3-pA).	This work
pSL1003	Mammalian P_{hCMV}-driven expression vector for NanoLuc-P2A-mINS-mRNA containing MCP-specific poly(A)-surrogate with 16 tandem MS2-box repeats (P_{hCMV}-NanoLuc-P2A-mINS-$(MS2\text{-}box)_{16}$-HHR-pA).	This work

pSL1014	Mammalian P_{hCMV}-driven constitutive BCR-ABL expression vector (P_{hCMV}-BCR-ABL-pA).	This work
pSL1032	AAV vector for P_{hCMV}-driven constitutive $(GNCR)_3$-NSP3 expression (P_{hCMV}-$(GNCR)_3$-NSP3-pA).	This work
pSL1042	AAV vector for P_{hCMV}-driven constitutive MCP-$(NS3a)_3$ expression (P_{hCMV}-MCP-$(NS3a)_3$-pA).	This work
pSL1045	Mammalian P_{hCMV}-driven constitutive BCR expression vector (P_{hCMV}-BCR-pA).	This work
pSL1046	Mammalian P_{hCMV}-driven constitutive ABL1 expression vector (P_{hCMV}-ABL1-pA).	This work
pSL1078	Mammalian P_{hCMV}-driven constitutive L7Ae-EGFP expression vector (P_{hCMV}-L7Ae-EGFP-pA).	This work
pSL1079	Mammalian P_{hCMV}-driven constitutive MCP-$(Coh2)_2$ expression vector (P_{hCMV}-MCP-$(Coh2)_2$-pA).	This work
pSL1080	Mammalian P_{hCMV}-driven constitutive MCP-$(Coh2)_3$ expression vector (P_{hCMV}-MCP-$(Coh2)_3$-pA).	This work
pSL1083	Mammalian P_{hCMV}-driven constitutive 3xFLAG-MCP-NSP3 expression vector (P_{hCMV}-3xFLAG-MCP-NSP3-pA).	This work
pSL1084	Mammalian P_{hCMV}-driven constitutive 3xFLAG-MCP expression vector (P_{hCMV}-3xFLAG-MCP-pA).	This work
pSL1085	P_{T7}-driven expression vector for *in vitro* transcription of MCP-$(NS3a)_3$ mRNA containing stabilized 5'-UTR, 3'-UTR and an extended poly(A) signal (P_{T7}-5'UTR*-MCP-$(NS3a)_3$-3'UTR*-A_{92}).	This work
pSL1091	P_{T7}-driven expression vector for *in vitro* transcription of SEAP mRNA containing an extended poly(A) signal (P_{T7}-MCP-$(NS3a)_3$-A_{92}-pA).	This work
pSL1093	Mammalian P_{hCMV}-driven constitutive 3xFLAG-MCP-NS3a expression vector (P_{hCMV}-3xFLAG-MCP-NS3a-pA).	This work

pSL1096	Mammalian P_{hCMV}-driven constitutive MCP-CIB1 expression vector (P_{hCMV}-MCP-CIB1-pA).	This work
pSL1097	Mammalian P_{hCMV}-driven constitutive MCP-FRB expression vector (P_{hCMV}-MCP-FRB-pA).	This work
pSL1098	Mammalian P_{hCMV}-driven constitutive FKBP-NSP3 expression vector (P_{hCMV}-FKBP-NSP3-pA).	This work
pSL1099	Mammalian P_{hCMV}-driven constitutive DocS-mCherry expression vector (P_{hCMV}-DocS-mCherry-pA).	This work
pSL1101	Mammalian P_{hCMV}-driven constitutive 3xFLAG-MCP-ABI(iDab)$_3$ expression vector (P_{hCMV}-3xFLAG-MCP-ABI(iDab)$_3$-pA).	This work
pSL1102	Mammalian P_{hCMV}-driven constitutive 3xHA-CCmut3-NSP3 expression vector (P_{hCMV}-3xHA-CCmut3-NSP3-pA).	This work
pSL1284	Mammalian P_{hCMV}-driven expression vector for SEAP-mRNA containing MCP-specific poly(A)-surrogate with 12 tandem MS2-box repeats (P_{hCMV}-SEAP-(MS2-box)$_{12}$-HHR-pA).	This work
pSL1308	Mammalian P_{hCMV}-driven expression vector for EGFP-mRNA containing MCP-specific poly(A)-surrogate with 24 tandem MS2-box repeats (P_{hCMV}-EGFP-(MS2-box)$_{24}$-HHR-pA).	This work
pSL1310	Mammalian P_{hCMV}-driven expression vector for SEAP-mRNA containing 12 tandem MS2-box repeats in the 3'-UTR (P_{hCMV}-SEAP-(MS2-box)$_{12}$-pA).	This work
pSL1311	Mammalian P_{hCMV} -driven constitutive MCP-DocS expression vector (P_{hCMV}-MCP-DocS-pA).	This work
pSL1314	SB100X-specific transposon containing constitutive expression units for BlastR and SEAP (ITR-P_{hCMV}-SEAP-pA:P_{SV40}-BlastR-pA-ITR).	This work

pSL1315	Mammalian P_{hCMV} -driven constitutive PABP-MCP expression vector (P_{hCMV}-PABP-MCP-pA).	This work
pSL1316	Mammalian P_{hCMV} -driven constitutive MCP-eIF4E expression vector (P_{hCMV}-MCP-eIF4E-pA).	This work
pSL1331	Mammalian P_{hCMV}-driven expression vector for shRNA-216-repressible MCP-specific SEAP-mRNA (P_{hCMV}-SEAP-(MS2-box)$_8$- BS(shRNA-216)$_2$-pA).	This work
pSL1429	Mammalian P_{hCMV}-driven constitutive eIF4G expression vector (P_{hCMV}-eIF4G-pA).	This work
pSL1430	Mammalian P_{hCMV}-driven constitutive eIF4E expression vector (P_{hCMV}-eIF4E-pA).	This work
pSL1431	Mammalian P_{hU6}-driven expression vector for eIF4G-specific shRNA (P_{hU6}-shR-eIF4G).	This work
pSL1432	Mammalian P_{hU6}-driven expression vector for eIF4E-specific shRNA (P_{hU6}-shR-eIF4E).	This work
pSL1470	Mammalian P_{hCMV}-driven constitutive PABP expression vector (P_{hCMV}-PABP-pA).	This work
pSL1471	Mammalian P_{hCMV}-driven constitutive NSP3 expression vector (P_{hCMV}-NSP3-pA).	This work
pSLM54	Mammalian P_{MusAFP}-driven NanoLuc expression vector (P_{MusAFP}-NanoLuc-pA).	This work
pSLM97	Mammalian P_{hCMV}-driven constitutive expression vector for SEAP-mRNA with self-cleavable polyA (P_{hCMV}-SEAP-HHR-pA).	This work
pDJ55	Mammalian $P_{hEF1\alpha}$-driven constitutive L7Ae-eIF4E^{K119A} expression vector ($P_{hEF1\alpha}$-L7Ae-eIF4E^{K119A}-pA).	This work
pLYL47	Mammalian P_{hCMV}-driven constitutive MCP-VPg expression vector. (P_{hCMV}-MCP-VPg-pA).	This work
pPW2	Mammalian P_{hCMV}-driven constitutive GAI-NSP3 expression vector (P_{hCMV}-GAI-NSP3-pA).	This work

pPW3	Mammalian P_{hCMV}-driven constitutive L7Ae-(ABI)$_3$ expression vector (P_{hCMV}-L7Ae-(ABI)$_3$-pA).	This work
pPW4	Mammalian P_{hCMV}-driven constitutive NSP3-(PYL)$_3$ expression vector (P_{hCMV}-NSP3-(PYL)$_3$-pA).	This work
pPW14	Mammalian P_{hCMV}-driven constitutive GAI-L7Ae expression vector (P_{hCMV}-GAI-L7Ae-pA).	This work
pPW17	Mammalian P_{hCMV}-driven constitutive NSP3-GID expression vector (P_{hCMV}-NSP3-GID-pA).	This work
pPW21	Mammalian P_{hCMV}-driven expression vector for reporter mRNA containing 24 tandem C/D-box repeats placed downstream of SEAP- and upstream of NanoLuc-coding regions (P_{hCMV}-SEAP-(C/Dbox)$_{24}$-NanoLuc-pA).	This work
pPW22	Mammalian P_{hCMV}-driven constitutive ABI-MCP expression vector (P_{hCMV}-ABI-MCP-pA).	This work
pPW23	Mammalian P_{hCMV}-driven constitutive MCP-GID1 expression vector (P_{hCMV}-MCP-GID1-pA).	This work
pPW24	Mammalian P_{hCMV}-driven constitutive (PYL)$_3$-NSP3 expression vector (P_{hCMV}-(PYL)$_3$-NSP3-pA).	This work
pQX183	Mammalian P_{hCMV}-driven constitutive mCherry expression vector (P_{hCMV}-mCherry-pA).	This work
pQZ8	Mammalian P_{hCMV}-driven expression vector for SEAP mRNA containing 4 tandem C/D-box repeats in the 5'-UTR (P_{hCMV}-(C/Dbox)$_4$-SEAP-pA).	This work

Abbreviations (Corresponding sequences or provided in Supplementary Information Table S3):

2CaM, calmodulin variant [5]; 3'UTR*, stabilized synthetic 3'-untranslated region; **3xFLAG**, polypeptide epitope containing three Flag repeats; **3xHA**, human influenza hemagglutinin tag; 5'UTR*, stabilized synthetic 5'-untranslated region; λN, bacteriophage λ-derived N-peptide [3]; **AAV**, adeno-associated virus; **ABI**, abscisic acid-responsive PYL1-binding protein[6]; **ABL**, tyrosine-protein kinase encoded by the human *ABL1* gene on chromosome 9; **ABl(iDab)**, intracellular antibody domain targeting ABL1 [7]; **Aff6_V18FΔN**, DrBPhP-specific affibody[8]; **ANR**, *apo* NS3a reader [9]; **mBax/hBax**, murine or human BCL2-associated X pro-apoptotic protein (NCBI-IDs: NM_007527, NM_001291428); **BCR**, Breakpoint cluster region protein encoded by the human *BCR* gene on chromosome 22; **BCR-ABL**, chronic myelogenous leukemia (CML)-specific oncoprotein produced by chromosomal translocation and genetic fusion of *BCR* and *ABL1* genes; **Bcl-XL**, B-cell lymphoma-extra large[10]; **BFP**, blue fluorescent protein; **BlastR,** gene conferring Blasticidin resistance; **BS(shRNA-216)**, binding site for shRNA-216; **CAAX**, prenylation site recognized by farnesyltransferase and geranylgeranyltransferase (amino acids: KMSKDGKKKKKKSKTKCVIM); **Cap8,** adeno-associated virus type 8 Cap protein; **CCmut3,** version 3 mutant of a BCR-specific coiled-coil domain [11]; **C/Dbox,** L7Ae-specific RNA aptamer (RNA sequence: 5'-GGGCGUGAUCCGAAAGGUGACCC-3'); **CIB1,** cryptochrome-interacting basic helix-loop-helix[12]; **Coh2**, *Clostridium thermocellum* cohesin[13]; **Cry2**, *Arabidopsis thaliana* cryptochrome 2 protein[12]; **DNCR2**, danoprevir/NS3a complex reader[14]; **DocS**, *Clostridium thermocellum* cellulose S derived dockerin[13]; **DrBPhP**, *Deinococcus radiodurans* BV-binding bacterial phytochrome[8]; **E2A,** early region 2A gene; **E4**, early region 4 gene, **EGFP**, enhanced green fluorescent protein; **eIF4E^{K119A}**, synthetic mutant of eukaryotic initiation factor 4E[15]; **eIF4G**, C-terminal fragment of eukaryotic initiation factor 4E[15]; **ERK2**, extracellular regulated protein kinase 2 (NCBI-ID: NM_138957); **FKBP**, FK506-binding protein[16]; **FLAG**, Flag® (DYKDDDDK) tag; **Fluc**, firefly luciferase [17]; **FRB,** FKBP-rapamycin binding (FRB) domain of the mammalian target of rapamycin (mTOR) kinase [16]; **GAI,** gibberellin insensitive[6]; **GID1**, gibberellin insensitive dwarf1[6]; **GNCR**, grazoprevir/NS3a complex reader [14]; **HHR**, λN-repressible hammerhead ribozyme (Env140/nutR/H6a)[3]; **ITR**, SB100X-specific inverted terminal repeats; **L7Ae**, archaeal ribosomal protein binding to the C/Dbox RNA kink-turn (K-turn) motif.[18]; **LaG16**, high-affinity anti-EGFP nanobody [19]; **LaM8_AK47**, light-inducible LaM8-AK47 nanobody[20]; **LD1**, lead design 1- a synthetic protein derived from rat syntaxin 6[10]; **LD3**, lead design 3- a synthetic protein derived from human focal adhesion targeting domain of human apolipoprotein E4[10]; **M13**, calmodulin-binding peptide[5]; **MALAT1**, metastasis-associated lung adenocarcinoma transcript 1 long non-coding RNA [21]; **mCherry**, mushroom coral red fluorescence protein; **MCP**, bacteriophage MS2 coat protein (GenBank: ASW25882.1); **MCS**, multiple cloning site; **mINS**, modified furin-cleavable insulin variant [22]; **MS2-box**, MCP-specific RNA

aptamer C-variant (RNA-sequence: 5'-UGAGGAUCACCCA-3'); **NanoLuc,** secreted *Oplophorus gracilirostris*-derived NanoLuc® luciferase[16]; **NES**, mammalian nuclear export signal; **NLS**, mammalian nuclear localization signal; **NS3a**, solubility optimized catalytically active hepatitis C virus protease[14]; **NS3a(H1)**, catalytically active NS3a variant[9]; **NSP3**, bovine rotavirus strain RF nonstructural protein 3 (UniProtKB/Swiss-Prot: Q86504.1); **P2A,** porcine teschovirus derived ribosome skipping sequence optimized for bicistronic expression in mammalian cells[17]; **pA**, poly-adenylation signal poly(A); **A_{92},** synthetic polyA signal containing 92 adenosine bases; **PABP**, poly(A) binding protein (NCBI-ID: XP_004402403.1); **P_{CAG}**, synthetic mammalian chimeric promoter containing cytomegalovirus early enhancer element and chicken β-actin promoter; **pE59**, DARPin targeting phosphorylated ERK2[23]; **P_{hCMV}**, human cytomegalovirus immediate early promoter; **$P_{hCMV*-1}$**, tetracycline-responsive promoter containing a TetR-specific heptameric *tetO* operator sequence ($tetO_7$-$P_{hCMVmin}$)[2]; **$P_{hCMVmin}$**, minimal version of P_{hCMV}; **$P_{hEF1\alpha}$**, human elongation factor 1α promoter; **P_{hU6}**, human U6 promoter; **P_{MusAFP}**, *Mus musculus* AFP-inducible promoter (NCBI-ID: NC_000071); P_{RPBSA}, constitutive synthetic mammalian promoter [17]; **P_{SV40}**, simian virus 40 promoter; **P_{T7},** promoter activated by the T7 bacteriophage RNA polymerase; **PuroR,** gene conferring puromycin resistance; **PYL**, pyrabactin resistance (PYR)-like protein[6]; **Rep2,** adeno-associated virus type 2 Rep protein; **SB100X**, optimized Sleeping Beauty transposase [24]; **SEAP**, human placental secreted alkaline phosphatase; **shR-eIF4G**, short hairpin RNA specific for endogenous eukaryotic initiation factor 4G (RNA-sequence: 5'-GCCCUUGUAGUGACCUUAGAACUCGAGUUCUAAGGUCACUACAAGGGC-3')[25] ; **shR-eIF4E**, short hairpin RNA specific for endogenous eukaryotic initiation factor 4E (RNA-sequence: 5'-CGGCUGAUCUCCAAGUUUGAUCUCGAGAUCAAACUUGGAGAUCAGCCG-3')[25]; **shR-EGFP**, short hairpin RNA specific for EGFP (RNA-sequence: 5'-CGGCAAGCUGACCCUGAAGUUCCUCGAGGAACUUCAGGGUCAGCUUGCCG-3'); **shRNA-216**, short hairpin RNA no. 216 (RNA-sequence: 5'-CCGGUAAUCUCAGCUGGCAACUGUGACUCGAGUCACAGUUGCCAGCUGAGAUUAUUUUUG-3'); **SP**, secretory peptide; **TetR**, *Escherichia coli* Tn10-derived tetracycline-dependent repressor of the tetracycline resistance gene; **$tetO_7$**, TetR-specific heptameric operator sequence; **T_M**, N-terminal transmembrane domain; **UTR**, untranslated region; **VA,** adenovirus virus-associated gene; **VP64**, tetrameric core of herpes simplex virus-derived transactivation domain; **VPg**, caliciviral VPg protein [26]; **ZeoR**, gene conferring zeocin resistance.

References:

1. Mao, T. *et al.* Long-Range Neuronal Circuits Underlying the Interaction between Sensory and Motor Cortex. *Neuron* **72**, 111–123 (2011).

2. Fussenegger, M., Moser, S., Mazur, X. & Bailey, J. E. Autoregulated Multicistronic Expression Vectors Provide One-Step Cloning of Regulated Product Gene Expression in Mammalian Cells. *Biotechnol Progr* **13**, 733–740 (1997).

3. Ausländer, S. *et al.* A general design strategy for protein-responsive riboswitches in mammalian cells. *Nat Methods* **11**, 1154–1160 (2014).

4. Shao, J. *et al.* Smartphone-controlled optogenetically engineered cells enable semiautomatic glucose homeostasis in diabetic mice. *Sci Transl Med* **9**, eaal2298 (2017).

5. Palmer, A. E. *et al.* Ca2+ Indicators Based on Computationally Redesigned Calmodulin-Peptide Pairs. *Chem Biol* **13**, 521–530 (2006).

6. Gao, Y. *et al.* Complex transcriptional modulation with orthogonal and inducible dCas9 regulators. *Nat Methods* **13**, 1043–1049 (2016).

7. Dixon, A. S., Constance, J. E., Tanaka, T., Rabbitts, T. H. & Lim, C. S. Changing the Subcellular Location of the Oncoprotein Bcr-Abl Using Rationally Designed Capture Motifs. *Pharmaceut Res* **29**, 1098–1109 (2012).

8. Kuwasaki, Y. *et al.* A red light–responsive photoswitch for deep tissue optogenetics. *Nat Biotechnol* 1–8 (2022) doi:10.1038/s41587-022-01351-w.

9. Cunningham-Bryant, D. *et al.* A Chemically Disrupted Proximity System for Controlling Dynamic Cellular Processes. *J Am Chem Soc* **141**, 3352–3355 (2019).

10. Giordano-Attianese, G. *et al.* A computationally designed chimeric antigen receptor provides a small-molecule safety switch for T-cell therapy. *Nat Biotechnol* **38**, 426–432 (2020).

11. Dixon, A. S. *et al.* Improved Coiled-Coil Design Enhances Interaction with Bcr-Abl and Induces Apoptosis. *Mol Pharmaceut* **9**, 187–195 (2012).

12. Kennedy, M. J. *et al.* Rapid blue-light–mediated induction of protein interactions in living cells. *Nat Methods* **7**, 973–975 (2010).

13. Wu, J. *et al.* A non-invasive far-red light-induced split-Cre recombinase system for controllable genome engineering in mice. *Nat Commun* **11**, 3708 (2020).

14. Foight, G. W. *et al.* Multi-input chemical control of protein dimerization for programming graded cellular responses. *Nat Biotechnol* **37**, 1209–1216 (2019).

15. Gregorio, E. D., Preiss, T. & Hentze, M. W. Translation driven by an eIF4G core domain in vivo. *EMBO J* **18**, 4865–4874 (1999).

16. Scheller, L., Strittmatter, T., Fuchs, D., Bojar, D. & Fussenegger, M. Generalized extracellular molecule sensor platform for programming cellular behavior. *Nat Chem Biol* **14**, 723–729 (2018).

17. Kowarz, E., Löscher, D. & Marschalek, R. Optimized Sleeping Beauty transposons rapidly generate stable transgenic cell lines. *Biotechnol J* **10**, 647–653 (2015).

18. Saito, H. *et al.* Synthetic translational regulation by an L7Ae–kink-turn RNP switch. *Nat Chem Biol* **6**, 71–78 (2010).

19. Morsut, L. *et al.* Engineering Customized Cell Sensing and Response Behaviors Using Synthetic Notch Receptors. *Cell* **164**, 780–791 (2016).

20. Gil, A. A. *et al.* Optogenetic control of protein binding using light-switchable nanobodies. *Nat Commun* **11**, 4044 (2020).

21. DiAndreth, B., Wauford, N., Hu, E., Palacios, S. & Weiss, R. PERSIST platform provides programmable RNA regulation using CRISPR endoRNases. *Nat Commun* **13**, 2582 (2022).

22. Ausländer, D. *et al.* A Synthetic Multifunctional Mammalian pH Sensor and CO2 Transgene-Control Device. *Mol Cell* **55**, 397–408 (2014).

23. Kummer, L. *et al.* Structural and functional analysis of phosphorylation-specific binders of the kinase ERK from designed ankyrin repeat protein libraries. *Proc National Acad Sci* **109**, E2248–E2257 (2012).

24. Mátés, L. *et al.* Molecular evolution of a novel hyperactive Sleeping Beauty transposase enables robust stable gene transfer in vertebrates. *Nat Genet* **41**, 753–761 (2009).

25. Huang, R. *et al.* CDKAL1 Drives the Maintenance of Cancer Stem-Like Cells by Assembling the eIF4F Translation Initiation Complex. *Adv. Sci.* **10**, 2206542 (2023).

26. Nakanishi, H. & Saito, H. Caliciviral protein-based artificial translational activator for mammalian gene circuits with RNA-only delivery. *Nat Commun* **11**, 1297 (2020).
